# Finite-time stability of the West Nile Virus model with Brownian motion

**DOI:** 10.1371/journal.pone.0354331

**Published:** 2026-08-06

**Authors:** Kangkang Chang

**Affiliations:** School of Mathematics and Statistics, Xinxiang University, Xinxiang, Henan, P.R. China; Universidad de Almeria, SPAIN

## Abstract

West Nile virus disease is an acute infectious illness caused by infection with the West Nile virus, which was first isolated from the blood of a febrile patient in the West Nile region of Uganda in 1937. In recent decades, West Nile fever has continued to expand across endemic regions globally, with no specific antiviral treatment or vaccine currently available for prevention. This study examines the finite-time stability of a stochastic West Nile virus model. Initially, the boundedness, existence and uniqueness of the solution are established. Furthermore, by constructing a Lyapunov function and utilizing the Gronwall inequality, a sufficient condition for the finite-time stability of the West Nile virus model is derived. Finally, numerical simulations are conducted to verify the theoretical results, and the influence of noise intensity, mortality rates and transmission rates on the finite-time stability of the model are analyzed. The results show that the lower the noise intensity and transmission rates, the greater the mortality rates, and the better the system can achieve finite-time stability.

## 1. Introduction

West Nile virus is a zoonotic disease caused by infection with the West Nile virus, primarily affecting birds, humans, and mammals such as horses and cattle. Birds serve as reservoir hosts for the virus, while humans are predominantly infected through the bites of infected mosquitoes. In 2023, cases of West Nile virus infection were reported in several countries across Europe and the United States, with Italy documenting a total of 336 cases of West Nile fever, resulting in 29 fatalities (http://m.cz.ithc.cn/article/463053). In 2024, multiple countries have also reported deaths attributed to West Nile virus. According to Israel¡¯s Health Ministry, 913 individuals have been confirmed infected with West Nile virus since the outbreak began in June, with a death toll reaching 70. As of September 11, Greece has recorded 162 cases of West Nile virus infection and 25 deaths. Additionally, recent West Nile virus infections have been reported in multiple European countries, including Greece, Italy, and Spain (http://m.cz.ithc.cn/article/463053). Currently, no specific antiviral treatment or vaccine is available to prevent the West Nile virus. The most straightforward and effective method to mitigate the risk of infection remains the avoidance of mosquito bites.

Mathematical models [1–8] provide an effective approach for analyzing the pathogenesis of West Nile virus. For instance, Bhowmick et al. [1] examined the influence of migratory bird movement patterns on the transmission dynamics of West Nile virus. Zhu et al. [3] developed a West Nile virus model incorporating seasonality and investigated its dynamics along with optimal control strategies. Maliyoni [5] analyzed a stochastic West Nile virus model, deriving the extinction threshold and identifying conditions that determine whether the disease persists or becomes extinct. Baafi and Hurford [7] explored the effects of temperature and rainfall on disease transmission, highlighting regional differences in vector control effectiveness over time.

Previous studies have primarily examined the dynamic behavior of West Nile virus using ordinary differential equation models. Concurrently, numerous researchers have developed diffusion model [9–21] tto further explore disease transmission. Wang et al. [9] investigated the long term behavior of a West Nile virus model with free boundaries. Lin and Zhu [10] incorporated the free boundaries into a reaction-diffusion West Nile model, introducing a spatio-temporal risk index. Xing et al. [12] analyzed a reaction-diffusion West Nile model with spatial heterogeneity, assessing the effects of spatial variation, dispersal rates, advection rate and bird recovery rates on species survival or extinction. Chang et al. [15] examined the dynamical behavior of a nonlocal diffusion West Nile virus model under spatial heterogeneity. Ge et al. [18]evaluated the impact of seasonal fluctuations on virus transmission. We also observe that mosquitoes and birds are highly susceptible to external disturbances. For instance: temperature, humidity, and rainfall can affect the reproductive and survival rates of mosquito vectors; insecticide spraying and vaccination campaigns face uncertainties in their implementation timing and efficacy; bird migration routes may alter disease transmission patterns. These factors collectively highlight the practical significance of investigating stochastic models to better capture the complexity and unpredictability of West Nile Virus dynamics. Up to now, there are many studies on stochastic epidemic models [22,23], but then, there are few papers on the dynamics of stochastic West Nile model models.

Moreover, these studies primarily focused on the long-term behavior of the disease, whereas in practical scenarios, controlling disease progression over a limited time frame is often of greater concern, namely, finite-time stability. Finite-time stability is an effective method for analyzing the transient response of systems, proposed by Dorato in 1961 [24]. If, under given initial condition constraints, the system state remains within a specified threshold during a predefined time interval, the system is considered finite-time stability. So far, some researchers have devoted efforts to the study of finite-time stability [24–29]. But, the finite-time stability analysis of stochastic West Nile Virus model has not yet been thoroughly investigated, which motivates the research presented in this paper. This study focuses on the finite-time stability of a West Nile virus model, investigating disease progression within a constrained period. The main contributions of this research are as follows: (1) To account for the influence of weather and temperature variations on disease transmission, stochastic noise is incorporated into the model. (2) A sufficient condition or the finite-time stability of the West Nile virus model is established. (3) Numerical simulations are conducted to validate the theoretical findings and examine the impact of noise intensity on finite-time stability.

This paper is structured as follows. Section 2 presents the development of the West Nile Virus model, where the boundedness, existence, and uniqueness of the solution are established. In Section 3, a Lyapunov function is constructed, and the Gronwall inequality is applied to derive a sufficient condition for the finite-time stability of the West Nile Virus model. Section 4 validates the results of Theorem 1.2 through numerical simulations. Finally, Section 5 concludes the paper with final remarks.

## 2. Model

The mortality rates of mosquito vectors and hosts are influenced by stochastic fluctuations in environmental factors such as temperature, humidity, and insecticide application. By modeling mortality rates as stochastic processes, the impact of these unpredictable disturbances on disease transmission dynamics can be more realistically captured. On the basis of Model (1.1) in reference [15], we perturb the parameters $\mu_{m}$ with $\mu_{m}-\varepsilon_{1}\eta_{1}(t)$ and $\mu_{b}$ with $\mu_{b}-\varepsilon_{2}\eta_{2}(t)$ [30], $dB_{i}=d\eta_{i}$, $\varepsilon_{i}(i=1,2)$ denote the noise intensity, $\eta_{i}$ represents the Gaussian white noise, then, we can obtain that the following model:


$$\begin{array}{c} \left\{ \begin{array}{c} \frac{dS_{m}}{dt}=\Lambda_{m}-\mu_{m}S_{m}-\frac{b_{1}\beta_{m}}{N_{b}}S_{m}I_{b}+\varepsilon_{1}S_{m}dB_{1}(t),\\ \frac{dI_{m}}{dt}=\frac{b_{1}\beta_{m}}{N_{b}}S_{m}I_{b}-\mu_{m}I_{m}+\varepsilon_{1}I_{m}dB_{1}(t),\\ \frac{dS_{b}}{dt}=\Lambda_{b}-\mu_{b}S_{b}-\frac{b_{1}\beta_{b}}{N_{b}}S_{b}I_{m}+\varepsilon_{2}S_{b}dB_{2}(t),\\ \frac{dI_{b}}{dt}=\frac{b_{1}\beta_{b}}{N_{b}}S_{b}I_{m}-\mu_{b}I_{b}-d_{b}I_{b}+\varepsilon_{2}I_{b}dB_{2}(t),\\ S_{m,0}=S_{m}(0),I_{m,0}=I_{m}(0),S_{b,0}=S_{b}(0),I_{b,0}=I_{b}(0), \end{array}\right. \end{array}$$ (1)

where $B_{i}(t),(i=1,2)$ defined on $\left(\Omega ,ℱ,\{{ℱ}_{t}{\}}_{t\geq 0},P\right)$, $\left(\Omega ,ℱ,\{{ℱ}_{t}{\}}_{t\geq 0},P\right)$ be a complete probability space. $W(t)=(S_{s}(t),I_{a}(t),S_{h},I_{h})\in R_{+}^{4}$, $\left\|W(t){\|}^{2}=S_{m}^{2}(t)+I_{m}^{2}(t)+S_{b}^{2}(t)+I_{b}^{2}(t\right)$, ‖·‖ denotes the norm. Let $N_{m}=S_{m}+I_{m}$ denotes the total number of mosquitoes, $N_{b}=S_{b}+I_{b}$ represents the total number of birds. All the parameters are bounded. The parameter descriptions are shown in Table 1.

**Table 1:** Parameters and their description.

Parameter	Description of parameter
$S_{m}$	the populations of uninfected female mosquitoes
$I_{m}$	the populations of infected female mosquitoes
$S_{b}$	the population of susceptible birds
$I_{b}$	the population of infected birds
$\Lambda_{m}$	the recruitment rates of susceptible mosquitoes
$\mu_{m}$	the natural death rates of mosquitos
$\beta_{m}$	the virus transmission rates from infected birds to uninfected mosquitoes
$N_{b}$	the recovery rate of birds
$\Lambda_{b}$	the recruitment rates of susceptible birds
$\mu_{b}$	the natural death rates of birds
$\beta_{b}$	the virus transmission rates from mosquitoes to birds
$d_{b}$	the disease-induced death rate of infected birds

## 3. Main results

### Existence and uniqueness of the solution

**Theorem 1.1** For the initial value $\left(S_{m,0},I_{m,0},S_{b,0},I_{b,0}\right)$, system (1) exists the unique global positive $\left(S_{m}(t),I_{m}(t),S_{b}(t),I_{b}(t)\right)$, and satisfies that $S_{m}(t)+I_{m}(t)+S_{b}(t)+I_{b}(t)<\infty$.

Proof. First, we prove $S_{m}(t)+I_{m}(t)+S_{b}(t)+I_{b}(t)<\infty$. By virtue of system (1), we have


$$\begin{array}{c} \begin{array}{c} d(S_{m}(t)+I_{m}(t)+S_{b}(t)+I_{b}(t))\\ =\Lambda_{m}-\mu_{m}(S_{m}+I_{m})+\Lambda_{b}-\mu_{b}S_{b}-(\mu_{b}+d_{b})I_{b}\\ \;+\varepsilon_{1}S_{m}dB_{1}(t)++\varepsilon_{1}I_{m}dB_{1}(t)+\varepsilon_{2}S_{b}dB_{2}(t)+\varepsilon_{2}I_{b}dB_{2}(t)\\ \leq \Lambda_{m}+\Lambda_{b}-min(\mu_{m},(\mu_{b}+d_{b}))\{S_{m}(t)+I_{m}(t)+S_{b}(t)+I_{b}(t)\}\\ \;+\varepsilon_{1}S_{m}dB_{1}(t)++\varepsilon_{1}I_{m}dB_{1}(t)+\varepsilon_{2}S_{b}dB_{2}(t)+\varepsilon_{2}I_{b}dB_{2}(t). \end{array} \end{array}$$

By virtue of the stochastic comparison theorem [31], we know that $S_{m}(t)+I_{m}(t)+S_{b}(t)+I_{b}(t)<\infty$.

Next, we show that there exists a unique solution for system (1). For the first equation of system (1), let $f(S_{m},I_{b})=\Lambda_{m}-\mu_{m}S_{m}-\frac{b_{1}\beta_{m}}{N_{b}}S_{m}I_{b}$, $g(S_{m})=\varepsilon_{1}S_{m}$, where $f(S_{m},I_{b})$ is the drift term, $g(S_{m})$ is the diffusion term.

Furthermore,


$$\begin{array}{c} \begin{array}{cc} & |\frac{\partial f}{\partial S_{m}}|=|-\mu_{m}-\frac{b_{1}\beta_{m}}{N_{b}}I_{b}|\leq \mu_{m}+b_{1}\beta_{m},\\ & |\frac{\partial f}{\partial I_{b}}|=|\frac{b_{1}\beta_{m}}{N_{b}}S_{m}|\leq \frac{b_{1}\beta_{m}}{N_{b}}N_{m},\\ & |\frac{\partial g}{\partial S_{m}}|=\varepsilon_{1}, \end{array} \end{array}$$

hence, the drift term and diffusion term of the first equation of system (1) satisfy the Lipschitz condition. For the other three equations in system (1), by applying the same approach, we can demonstrate that their drift terms and diffusion terms satisfy the Lipschitz conditions. As a result, there is a unique local solution on $t\in [0,\tau_{e})$, where $\tau_{e}$ is the explosion time.

Finally, we prove the uniqueness of the solution. Let *k*_0_ > 1 be sufficiently large. For each integer *k* > *k*_0_, define the stopping time


$$\begin{array}{c} \begin{array}{c} \tau_{k}=inf\{t\in [0,\tau_{e}]:min(S_{m}(t),I_{m}(t),S_{b}(t),I_{b}(t))\leq \frac{1}{k}\\ \;\;\;\;or\;max(S_{m}(t),I_{m}(t),S_{b}(t),I_{b}(t))\geq k\}. \end{array} \end{array}$$

Let inf∅=∞(the infimum of the empty set). $\tau_{k}$ is increasing as k→∞. Let $\tau_{\infty }=\lim_{k\to \infty }\tau_{k}$, then $\tau_{\infty }<\tau_{e}$ a.s.

Next, we prove that $\tau_{\infty }=\infty$ a.s. If the conclusion doesn’t hold, then there exists a number *T* > 0 and 0<ϵ<1 such that $\mathbb{P}\{\tau_{\infty }<\infty \}>\epsilon$. Therefore, there is an integer $k_{1}\geq k_{0}$ satisfy that $\mathbb{P}\{\tau_{k}<T\}\geq \epsilon ,\;k\geq k_{1}$.

Define the Lyapunov function as follows:


$$\begin{array}{c} \tilde{V}(W(t))=(S_{m}-1-\log S_{m})+(I_{m}-1-\log I_{m})+(S_{b}-1-\log S_{b})+(I_{b}-1-\log I_{b}). \end{array}$$ (2)

For equation (2), According to the Itô formula, we have


$$\begin{array}{c} \begin{array}{cc} d\tilde{V}(W(t))\;\;\;\; & =(1-\frac{1}{S_{m}})(\Lambda_{m}-\mu_{m}S_{m}-\frac{b_{1}\beta_{m}}{N_{b}}S_{m}I_{b})dt+\frac{1}{2}\varepsilon_{1}^{2}dt+\varepsilon_{1}(S_{m}-1)dB_{1}(t)\\ & \;+(1-\frac{1}{I_{m}})(\frac{b_{1}\beta_{m}}{N_{b}}S_{m}I_{b}-\mu_{m}I_{m})dt+\frac{1}{2}\varepsilon_{1}^{2}dt+\varepsilon_{1}(I_{m}-1)dB_{1}(t)\\ & \;+(1-\frac{1}{S_{b}})(\Lambda_{b}-\mu_{b}S_{b}-\frac{b_{1}\beta_{b}}{N_{b}}S_{b}I_{m})dt+\frac{1}{2}\varepsilon_{2}^{2}dt+\varepsilon_{2}(S_{b}-1)dB_{2}(t)\\ & \;+(1-\frac{1}{I_{b}})(\frac{b_{1}\beta_{b}}{N_{b}}S_{b}I_{m}-\mu_{b}I_{b}-d_{b}I_{b})dt+\frac{1}{2}\varepsilon_{2}^{2}dt+\varepsilon_{2}(I_{h}-1)dB_{2}(t)\\ & \leq \{\Lambda_{m}-\mu_{m}S_{m}+\mu_{m}+\frac{b_{1}\beta_{m}}{N_{b}}I_{b}+\varepsilon_{1}^{2}+\frac{b_{1}\beta_{m}}{N_{b}}S_{m}I_{b}-\mu_{m}I_{m}+\mu_{m}+\Lambda_{b}-\mu_{b}S_{b}\\ & \;-\frac{b_{1}\beta_{b}}{N_{b}}S_{b}I_{m}+\mu_{b}+\frac{b_{1}\beta_{b}}{N_{b}}I_{m}+\frac{b_{1}\beta_{b}}{N_{b}}S_{b}I_{m}-\mu_{b}I_{b}-d_{b}I_{b}+\mu_{b}+d_{b}+\varepsilon_{2}^{2}\}\\ & \;+\varepsilon_{1}(S_{m}-1)dB_{1}(t)+\varepsilon_{1}(I_{m}-1)dB_{1}(t)+\varepsilon_{2}(S_{b}-1)dB_{2}(t)+\varepsilon_{2}(I_{b}-1)dB_{2}(t). \end{array} \end{array}$$ (3)

According to the previous results, we have


$$\begin{array}{c} d\tilde{V}(W(t))\leq M+\varepsilon_{1}(S_{m}-1)dB_{1}(t)+\varepsilon_{1}(I_{m}-1)dB_{1}(t)+\varepsilon_{2}(S_{b}-1)dB_{2}(t)+\varepsilon_{2}(I_{b}-1)dB_{2}(t). \end{array}$$ (4)

We integrate both sides of (4) from 0 to $\tau_{k}\wedge T$ and take expectations


$$\begin{array}{c} E\tilde{V}(W(\tau_{k}\wedge T))\leq \tilde{V}(W(0))+MT. \end{array}$$

Let $\Omega_{k}=\{\tau_{k}\leq T\}$, we know that $P(\Omega_{k})\geq \epsilon$. Note that for any $\varpi \in \Omega_{k}$, there is at least one of $S_{a}(\tau_{k},\varpi )$, $I_{a}(\tau_{k},\varpi )$, $S_{h}(\tau_{k},\varpi )$, $I_{h}(\tau_{k},\varpi )$ equaling either *k* or $\frac{1}{k}$. Therefore


$$\begin{array}{c} \tilde{V}(W(\tau_{k}\wedge T))\geq (k-1-\log k)\wedge (\frac{1}{k}-1-\log\frac{1}{k}). \end{array}$$

Furthermore


$$\begin{array}{c} \begin{array}{cc} \tilde{V}(W(0))+MT\;\;\;\; & \geq E\{1_{\Omega_{k}}\tilde{V}(N(\tau_{k}\wedge T))\}\\ & \geq \epsilon \{(k-1-\log k)\wedge (\frac{1}{k}-1-\log\frac{1}{k})\}, \end{array} \end{array}$$

where $1_{\Omega_{k}}$ denotes the indicator function. As k→∞, we have


$$\begin{array}{c} \infty >\tilde{V}(W(0))+MT=\infty . \end{array}$$

This is a contradiction. Hence, we have $\tau_{\infty }=\infty$.

### Stability of system

Before proving that the system (1) is finite-time stability, according to references [26], we first give the following definition.

**Definition 1.1** There exists positive number *T*, *B*_1_, *B*_2_(where $B_{2}>B_{1}$) such that system (1) is finite-time stability, if $S_{m,0}+I_{m,0}+S_{b,0}+I_{b,0}\leq B_{1}$, then, for any t∈[0,T], we have


$$\begin{array}{c} 𝔼(S_{m}^{2}(t)+I_{m}^{2}(t)+S_{b}^{2}(t)+I_{b}^{2}(t))\leq B_{2}. \end{array}$$

**Remark 1** Under given initial condition constraints, the system state remains within a specified threshold during a predefined time interval, the system is considered finite-time stability.

Next, we will give and prove the conditions of finite-time stability for system (1).

**Theorem 1.2** If the following conditions hold


$$\begin{array}{c} 1+\varepsilon_{1}^{2}+b_{1}^{2}-2\mu_{m}>0,\beta_{m}^{2}+b_{1}^{2}+\varepsilon_{1}^{2}-2\mu_{m}>0,1+\varepsilon_{2}^{2}-2\mu_{b}>0,\beta_{b}^{2}+\varepsilon_{2}^{2}-2\mu_{b}-2d_{b}>0, \end{array}$$

then, system (1) is finite-time stability.

Proof. Define


$$\begin{array}{c} 𝕍(t)=S_{m}^{2}(t)+I_{m}^{2}(t)+S_{b}^{2}(t)+I_{b}^{2}(t). \end{array}$$

According to the Itô formula, we have


$$\begin{array}{c} \begin{array}{cc} d𝕍(t)= & \;\;\;\;2S_{m}(t)[\Lambda_{m}-\mu_{m}S_{m}-\frac{b_{1}\beta_{m}}{N_{b}}S_{m}I_{b}]dt+\varepsilon_{1}^{2}S_{m}^{2}(t)dt+2\varepsilon_{1}S_{m}^{2}(t)dB_{1}(t)\\ & \;\;\;\;+2I_{m}(t)[\frac{b_{1}\beta_{m}}{N_{b}}S_{m}I_{b}-\mu_{m}I_{m}]dt+\varepsilon_{1}^{2}I_{m}^{2}(t)dt+2\varepsilon_{1}I_{m}^{2}(t)dB_{1}(t)\\ & \;\;\;\;+2S_{b}(t)[\Lambda_{b}-\mu_{b}S_{b}-\frac{b_{1}\beta_{b}}{N_{b}}S_{b}I_{m}]dt+\varepsilon_{2}^{2}S_{b}^{2}(t)dt+2\varepsilon_{2}S_{b}^{2}(t)dB_{2}(t)\\ & \;\;\;\;+2I_{b}(t)[\frac{b_{1}\beta_{b}}{N_{b}}S_{b}I_{m}-\mu_{b}I_{b}-d_{b}I_{b}]dt+\varepsilon_{2}^{2}I_{b}^{2}(t)dt+2\varepsilon_{2}I_{b}^{2}(t)dB_{2}(t) \end{array} \end{array}$$ (5)

Furthermore, we have


$$\begin{array}{c} \begin{array}{cc} d𝕍(t)\leq & \;\;\;\;[\Lambda_{m}^{2}+S_{m}^{2}(t)-2\mu_{m}S_{m}^{2}(t)+\varepsilon_{1}^{2}S_{m}^{2}(t)]dt+[\beta_{m}^{2}I_{m}^{2}(t)+b_{1}^{2}S_{m}^{2}(t)\\ & \;\;\;\;-2\mu_{m}I_{m}^{2}(t)+\varepsilon_{1}^{2}I_{m}^{2}(t)]dt+[\Lambda_{b}^{2}+S_{b}^{2}(t)-2\mu_{b}S_{b}^{2}(t)+\varepsilon_{2}^{2}S_{b}^{2}(t)]dt\\ & \;\;\;\;+[\beta_{b}^{2}I_{b}^{2}(t)+b_{1}^{2}I_{m}^{2}(t)-2\mu_{b}I_{b}^{2}(t)-2d_{b}I_{b}^{2}(t)+\varepsilon_{2}^{2}I_{b}^{2}(t)]dt\\ & \;\;\;\;+2\varepsilon_{1}S_{m}^{2}(t)dB_{1}(t)+2\varepsilon_{1}I_{m}^{2}(t)dB_{1}(t)+2\varepsilon_{2}S_{b}^{2}(t)dB_{2}(t)+2\varepsilon_{2}I_{b}^{2}(t)dB_{2}(t). \end{array} \end{array}$$ (6)

For Equation (6), we can integrate from 0 to t∈[0,T] and take expectation


$$\begin{array}{c} \begin{array}{cc} 𝔼 & \;\;\;\;\;\;(S_{m}^{2}(t)+I_{m}^{2}(t)+S_{b}^{2}(t)+I_{b}^{2}(t))-(S_{m}^{2}(0)+I_{m}^{2}(0)+S_{b}^{2}(0)+I_{b}^{2}(0))\\ \leq & \;\;\;\;(\Lambda_{m}^{2}+\Lambda_{b}^{2})t+𝔼\int_{0}^{t}\{(1+\varepsilon_{1}^{2}+b_{1}^{2}-2\mu_{m})S_{m}^{2}(s)+(\beta_{m}^{2}+b_{1}^{2}+\varepsilon_{1}^{2}-2\mu_{m})I_{m}^{2}(s)\\ & \;\;\;\;+(1+\varepsilon_{2}^{2}-2\mu_{b})S_{m}^{2}(s)+(\beta_{b}^{2}+\varepsilon_{2}^{2}-2\mu_{b}-2d_{b})I_{b}^{2}(s)\}ds. \end{array} \end{array}$$ (7)

Due to the following conditions hold, $1+\varepsilon_{1}^{2}+b_{1}^{2}-2\mu_{m}>0$, $\beta_{m}^{2}+b_{1}^{2}+\varepsilon_{1}^{2}-2\mu_{m}>0$, $1+\varepsilon_{2}^{2}-2\mu_{b}>0$, $\beta_{b}^{2}+\varepsilon_{2}^{2}-2\mu_{b}-2d_{b}>0$, hence Equation (7) is equivalent to


$$\begin{array}{c} \begin{array}{cc} 𝔼 & \;\;\;\;\;\;(S_{m}^{2}(t)+I_{m}^{2}(t)+S_{b}^{2}(t)+I_{b}^{2}(t))\\ \leq & \;\;\;\;(S_{m}^{2}(0)+I_{m}^{2}(0)+S_{b}^{2}(0)+I_{b}^{2}(0))+(\Lambda_{m}^{2}+\Lambda_{b}^{2})t\\ & \;\;\;\;+M𝔼\int_{0}^{t}\{S_{m}^{2}(s)+I_{m}^{2}(s)+S_{m}^{2}(s)+I_{b}^{2}(s)\}ds, \end{array} \end{array}$$ (8)

where

$M=max\{1+\varepsilon_{1}^{2}+b_{1}^{2}-2\mu_{m},\beta_{m}^{2}+b_{1}^{2}+\varepsilon_{1}^{2}-2\mu_{m},1+\varepsilon_{2}^{2}-2\mu_{b},\beta_{b}^{2}+\varepsilon_{2}^{2}-2\mu_{b}-2d_{b}\}$. By virtue of the [32, Lemma 3.2, section 2.3], we can obtain


$$\begin{array}{c} \begin{array}{cc} 𝔼 & \;\;\;\;\;\;(S_{m}^{2}(t)+I_{m}^{2}(t)+S_{b}^{2}(t)+I_{b}^{2}(t))\\ \leq & \;\;\;\;\{(S_{m}^{2}(0)+I_{m}^{2}(0)+S_{b}^{2}(0)+I_{b}^{2}(0))+(\Lambda_{m}^{2}+\Lambda_{b}^{2})t\}e^{Mt}. \end{array} \end{array}$$ (9)

Due to t∈[0,T], we have


$$\begin{array}{c} \begin{array}{cc} 𝔼 & \;\;\;\;\;\;(S_{m}^{2}(t)+I_{m}^{2}(t)+S_{b}^{2}(t)+I_{b}^{2}(t))\\ \leq & \;\;\;\;\{(S_{m}^{2}(0)+I_{m}^{2}(0)+S_{b}^{2}(0)+I_{b}^{2}(0))+(\Lambda_{m}^{2}+\Lambda_{b}^{2})T\}e^{MT}:=B_{2}. \end{array} \end{array}$$ (10)

Therefore, system (1) is finite-time stability.

## 4. Numerical simulations

First, we set the parameter values as follows:

Let *T* = 2, *B*_1_ = 41, *B*_2_ = 544.752, $\left(S_{m,0},I_{m,0},S_{b,0},I_{b,0})=(4.5,2.5,3.5,1.5\right)$, according to Table 2, we can obtain the following conditions, $1+\varepsilon_{1}^{2}+b_{1}^{2}-2\mu_{m}>0$, $\beta_{m}^{2}+b_{1}^{2}+\varepsilon_{1}^{2}-2\mu_{m}>0$, $1+\varepsilon_{2}^{2}-2\mu_{b}>0$, $\beta_{b}^{2}+\varepsilon_{2}^{2}-2\mu_{b}-2d_{b}>0$.

**Table 2:** Value of parameter.

Parameter	Value
$\Lambda_{m}$	6.5 [15]
$\mu_{m}$	0.15 [15]
$\beta_{m}$	0.6 [5]
$\varepsilon_{1}$	0.25
*b*_1_	0.08
$N_{b}$	150 [15]
$\Lambda_{b}$	1.5 [15]
$\varepsilon_{2}$	0.15
$\beta_{b}$	0.88 [19,33]
$\mu_{b}$	0.2
$d_{b}$	0.12

### The effect of noise intensity on finite-time stability

**Fig 1:**
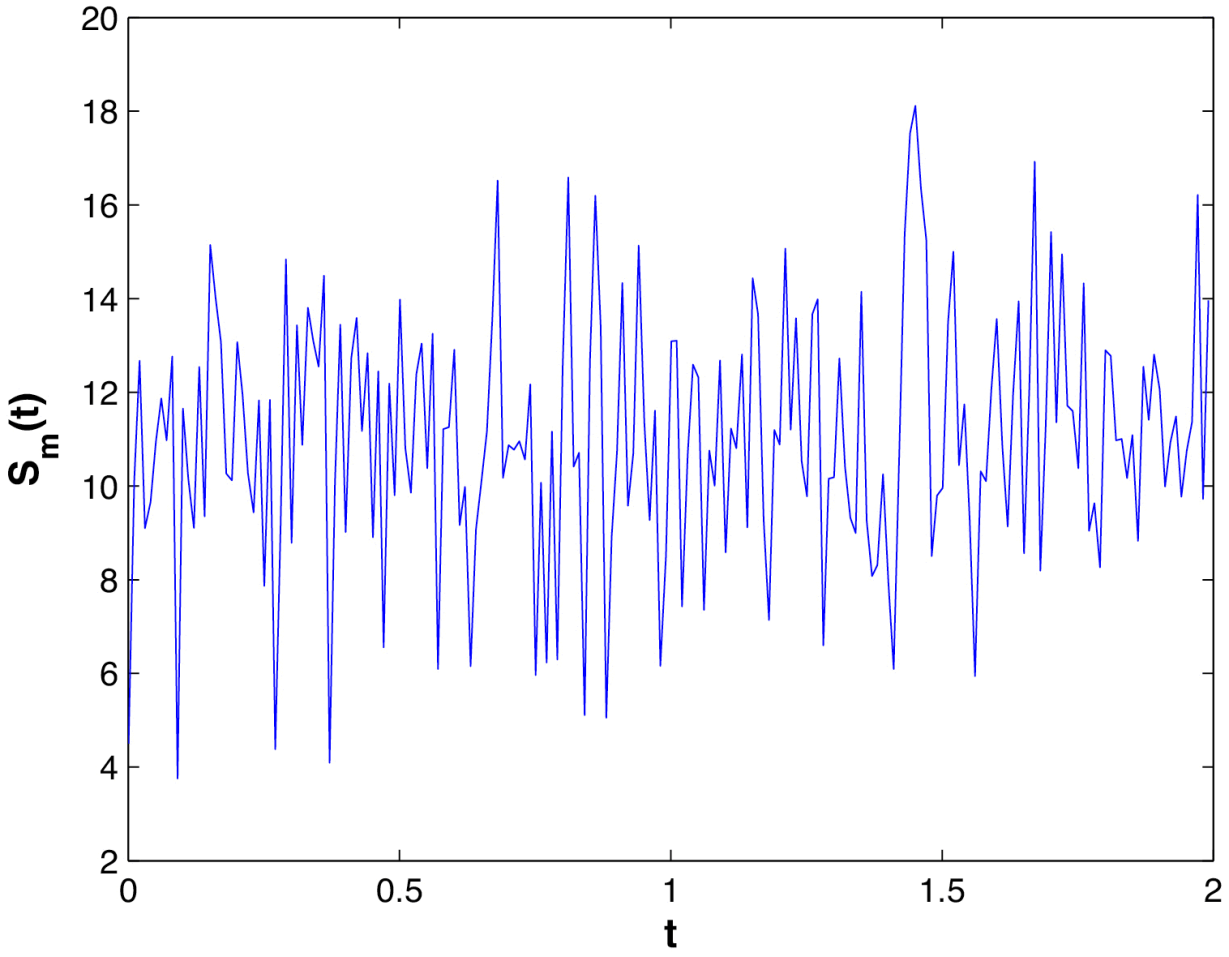
The evolution path of $S_{m}$ for moderate noise intensity.

**Fig 2:**
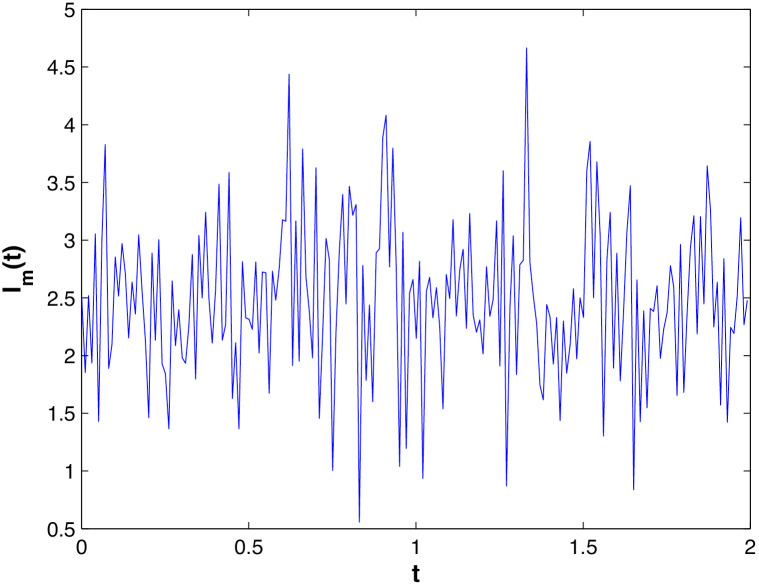
The evolution path of $I_{m}$ for moderate noise intensity.

**Fig 3:**
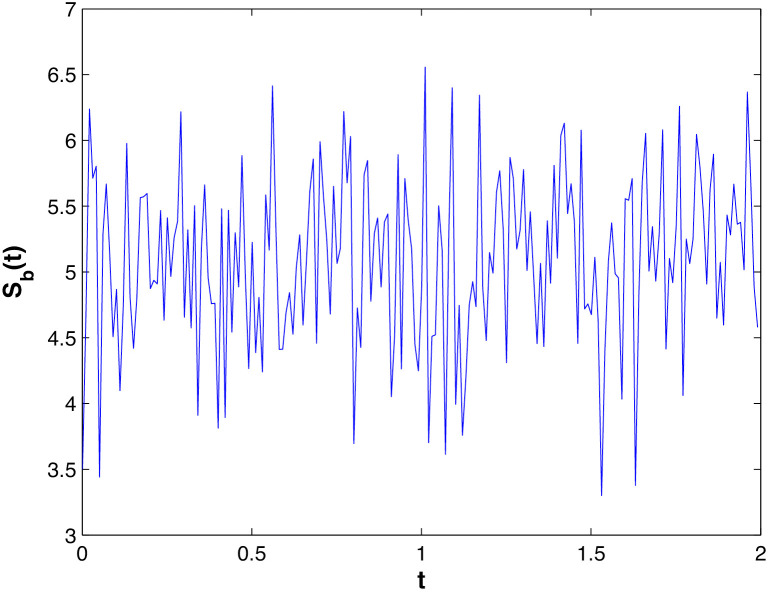
The evolution path of $S_{b}$ for moderate noise intensity.

**Fig 4:**
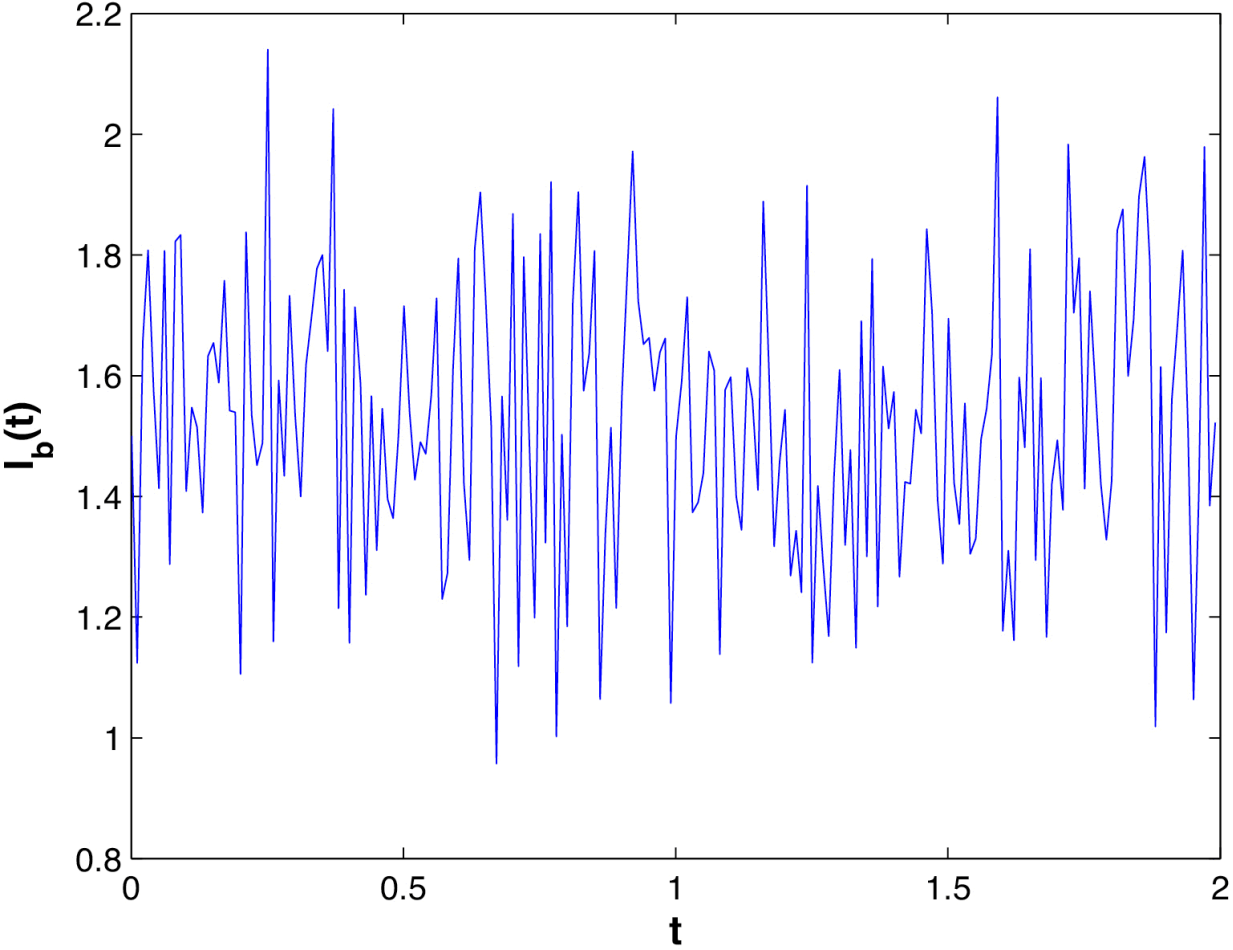
The evolution path of $I_{b}$ for moderate noise intensity.

**Fig 5:**
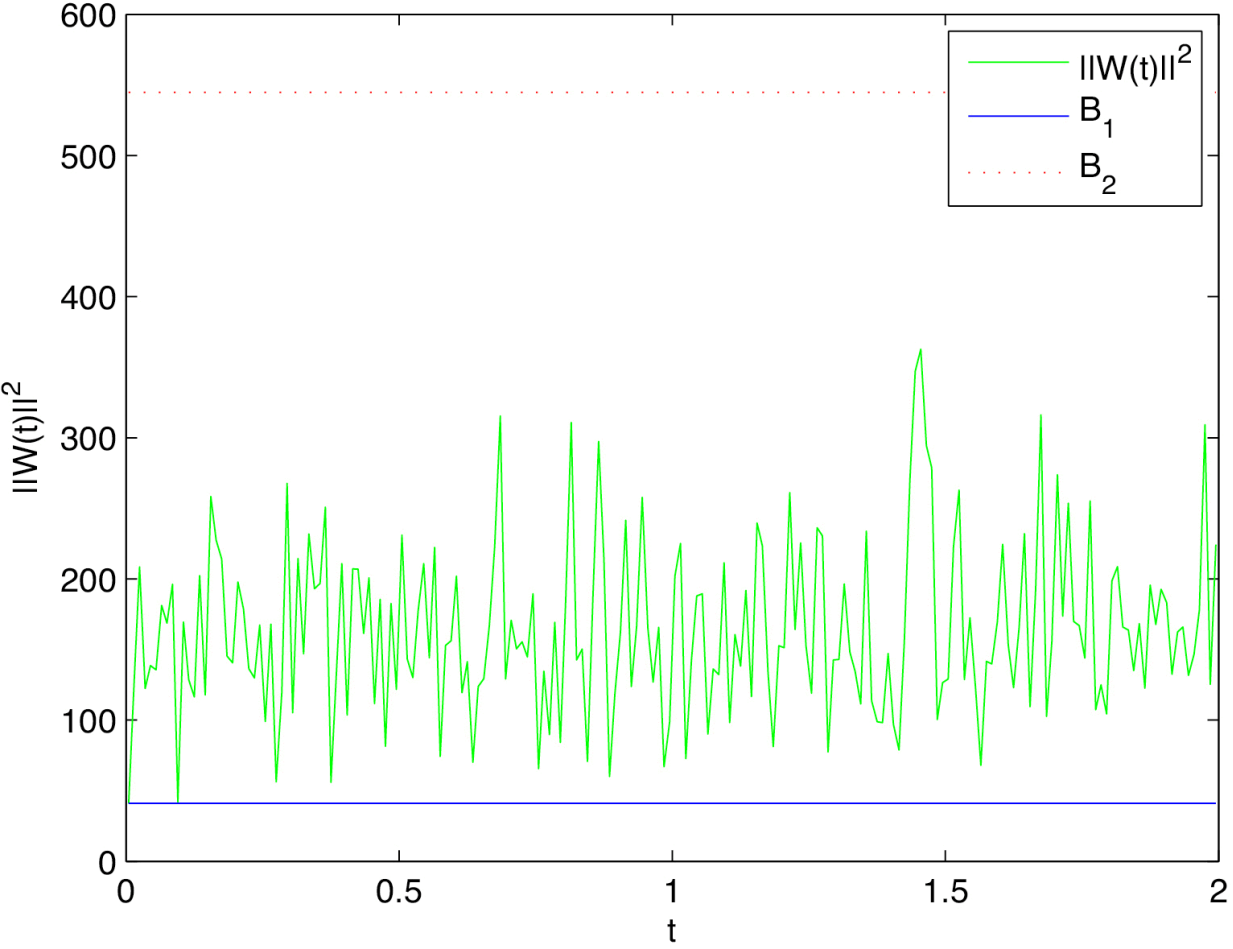
The state trajectories of system (1) for moderate noise intensity.

Based on the conditions of Theorem 1.2, it can be concluded that system (1) exhibits finite-time stability with respect to (2,41,544.752). In Figs 1–5, let $\varepsilon_{1}=0.25$, $\varepsilon_{2}=0.15$, the evolution trajectories of $S_{m},I_{m},S_{b},I_{b}$ and the state trajectories of the norm *W* (*t*) are presented.

**Fig 6:**
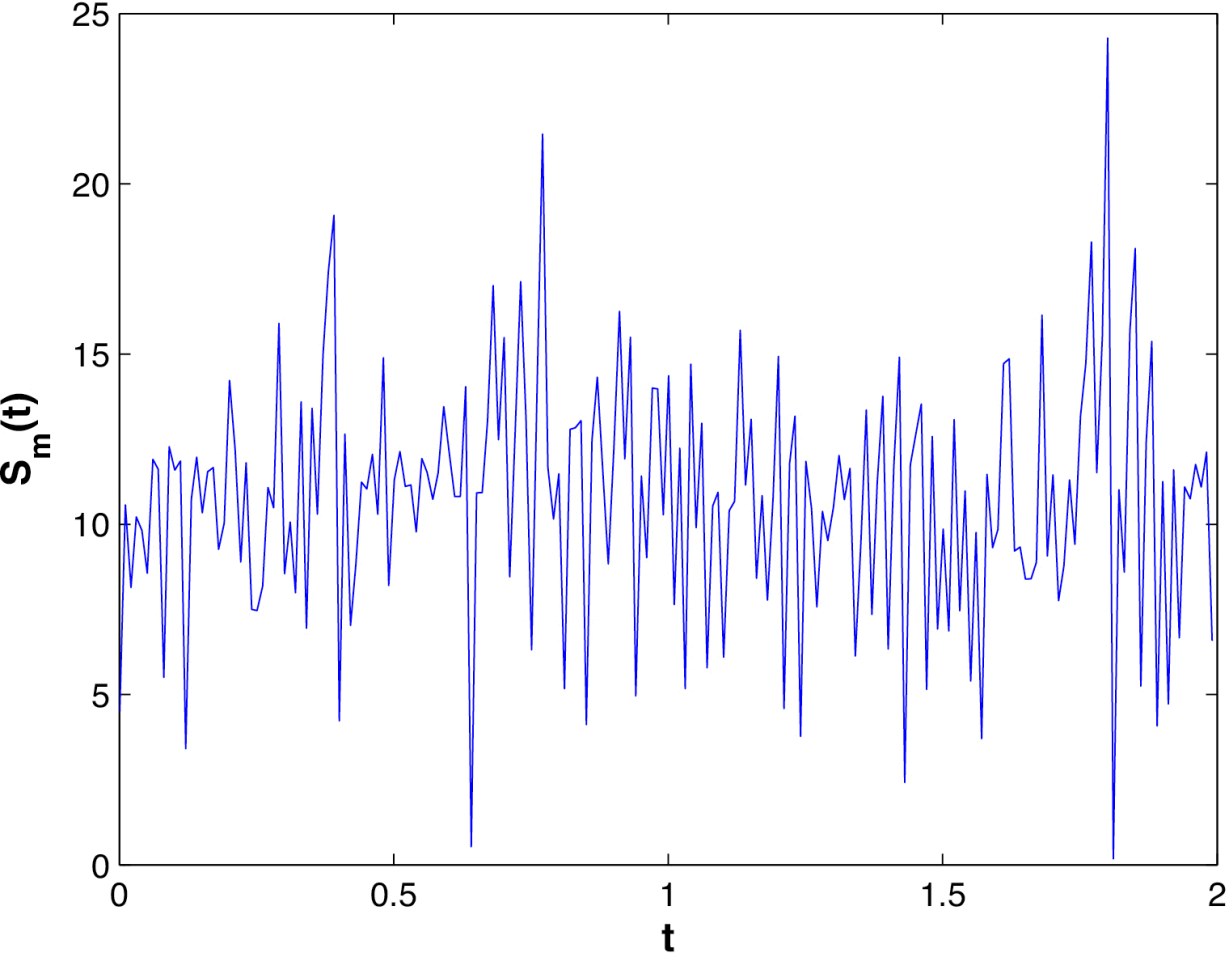
The evolution path of $S_{m}$ for high noise intensity.

**Fig 7:**
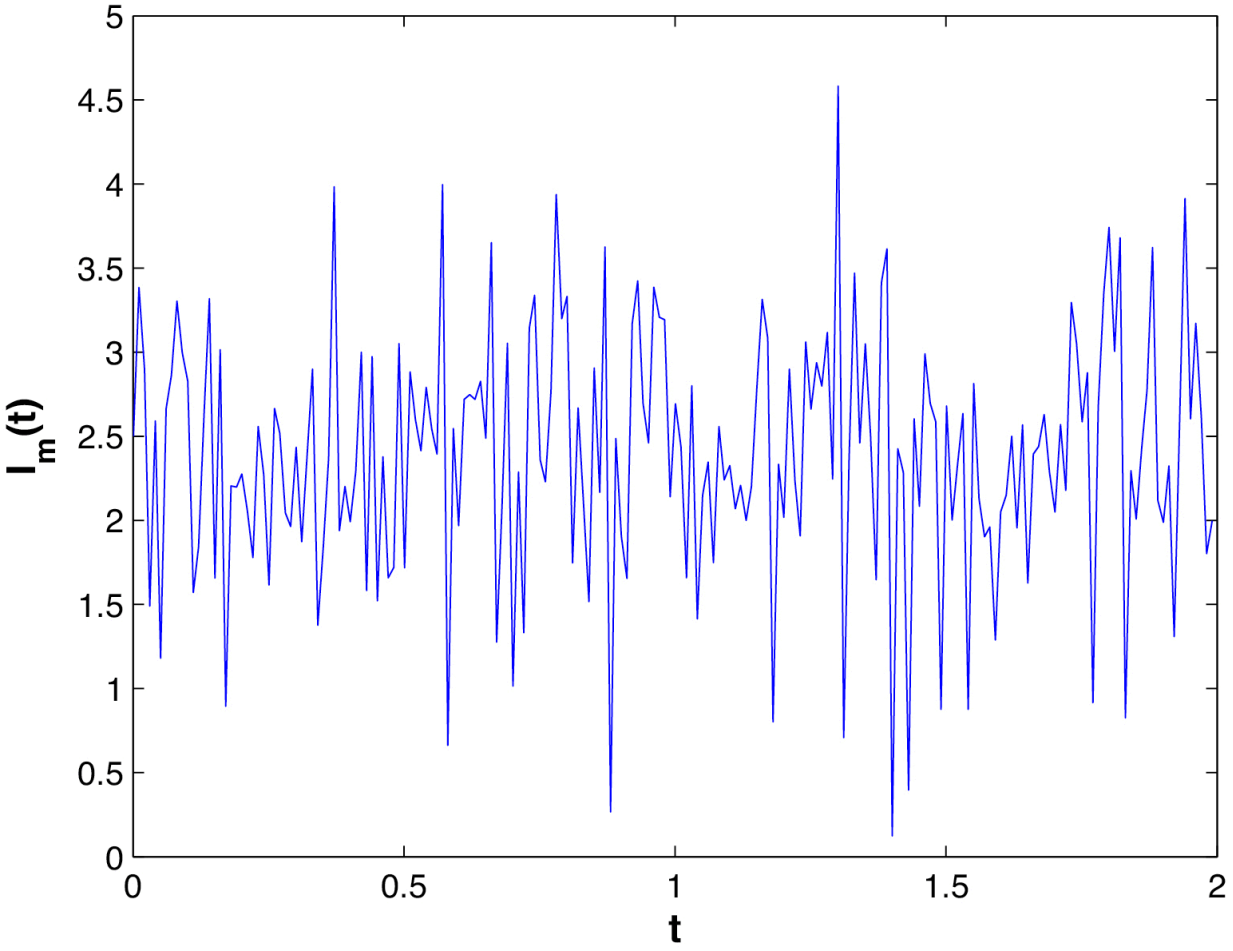
The evolution path of $I_{m}$ for high noise intensity.

**Fig 8:**
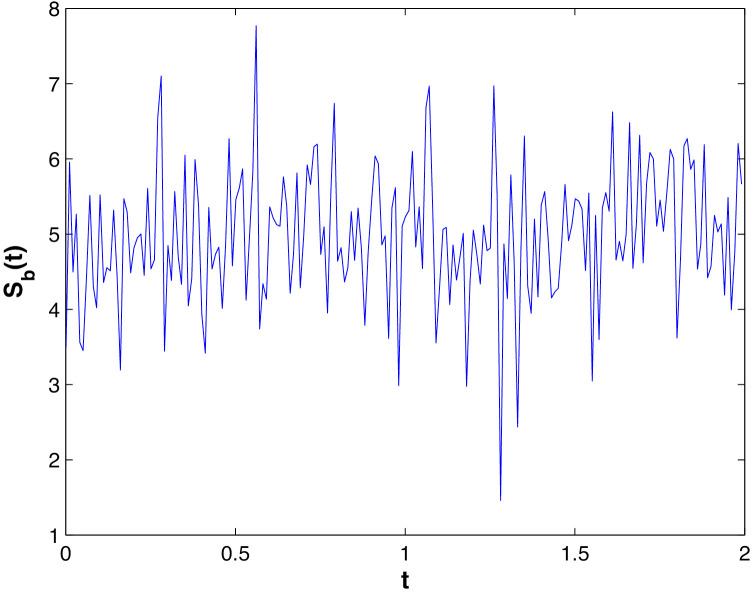
The evolution path of $S_{b}$ for high noise intensity.

**Fig 9:**
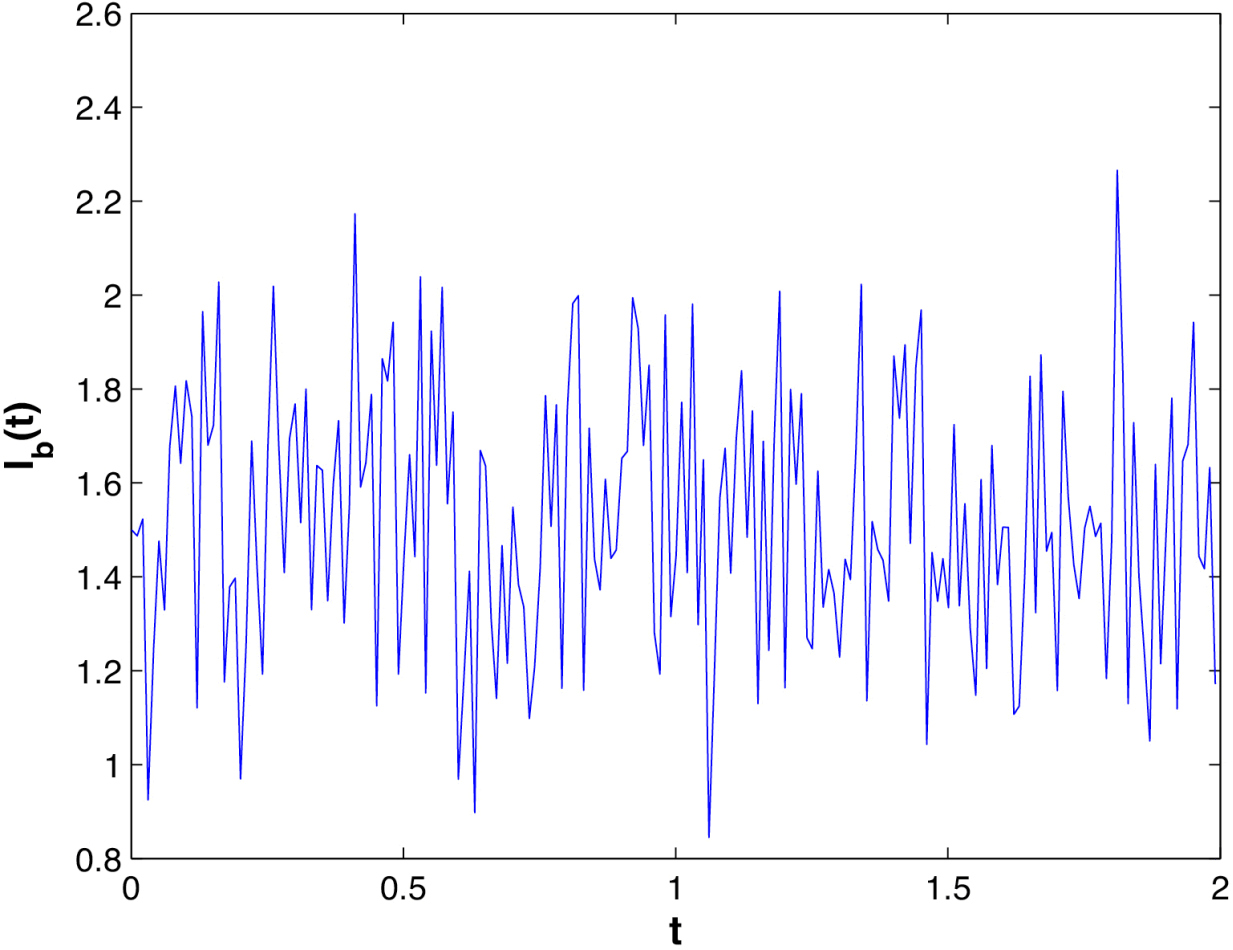
The evolution path of $I_{b}$ for high noise intensity.

**Fig 10:**
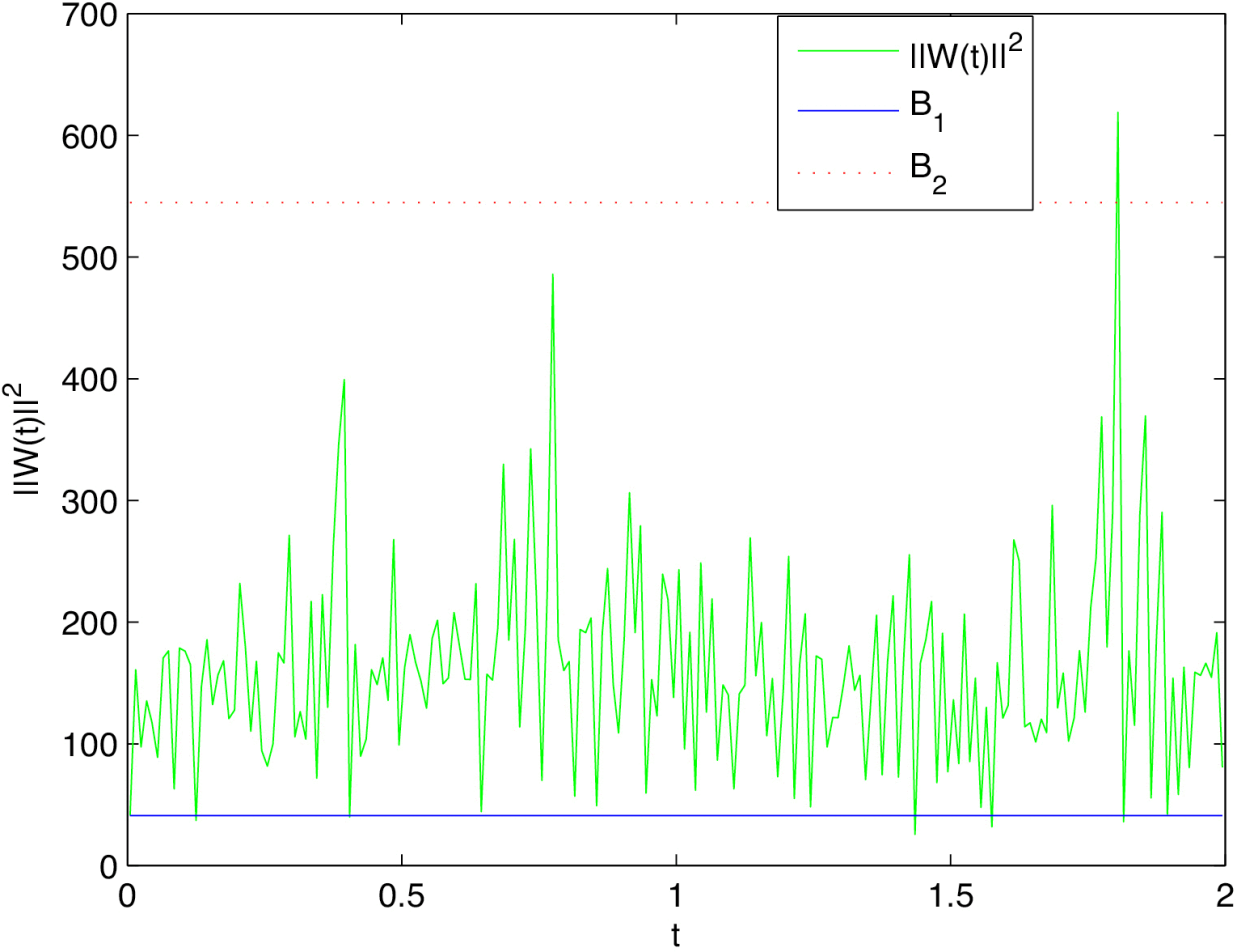
The state trajectories of system (1) for high noise intensity.

In Figs 6–10, let $\varepsilon_{1}=0.3$, $\varepsilon_{2}=0.2$, the evolution trajectories of $S_{m},I_{m},S_{b},I_{b}$ and the state trajectories of the norm *W* (*t*) are presented.

**Fig 11:**
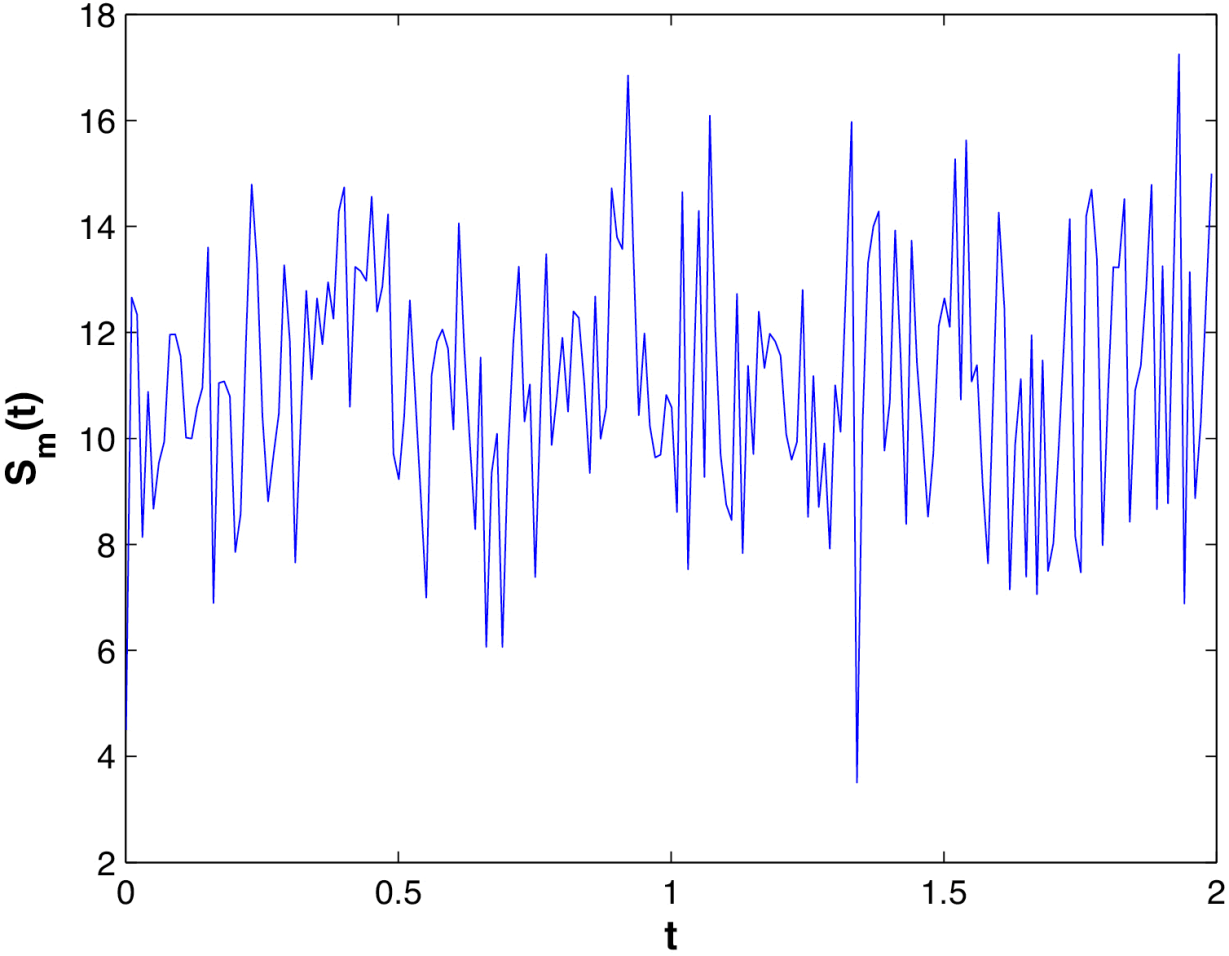
The evolution path of $S_{m}$ for low noise intensity.

**Fig 12:**
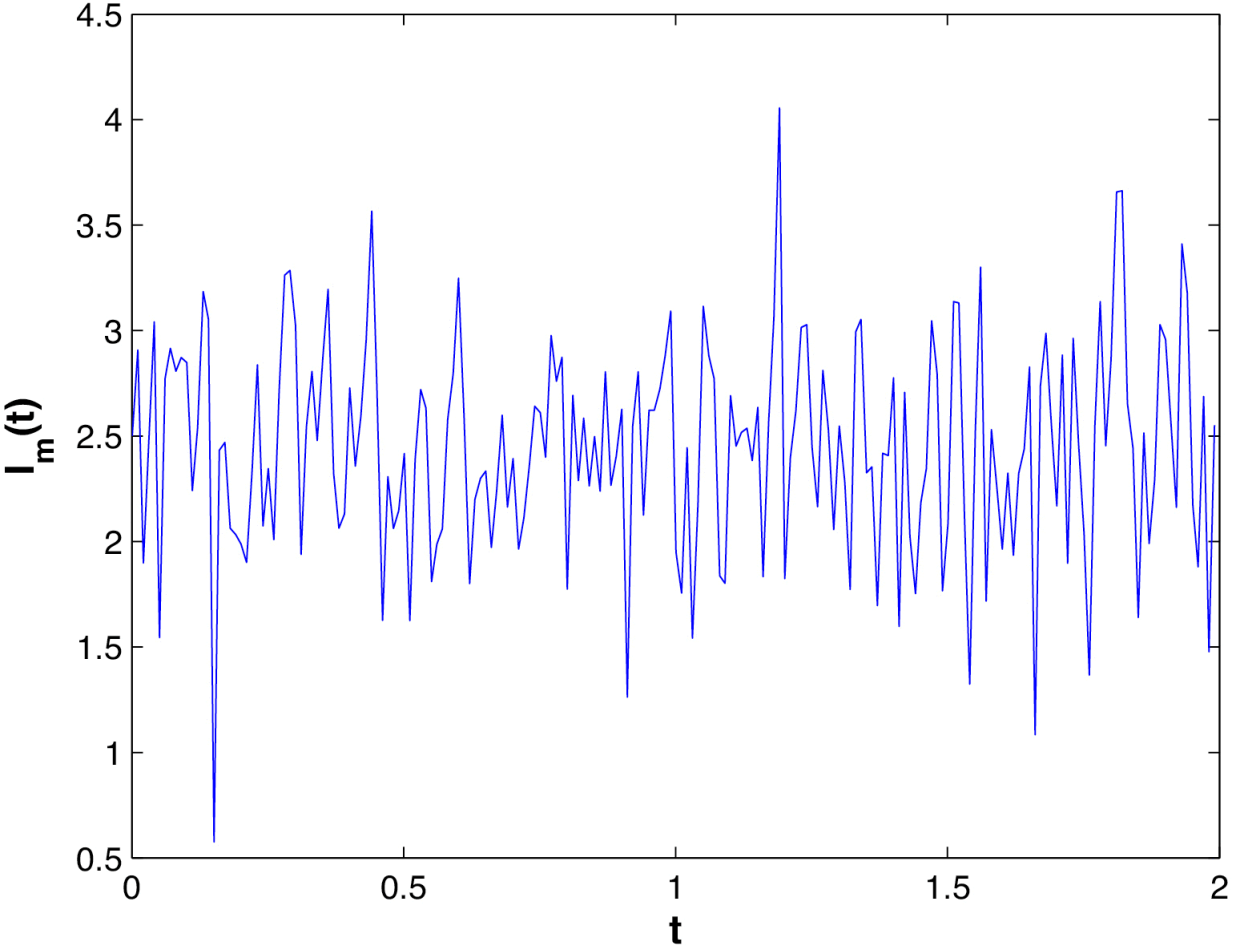
The evolution path of $I_{m}$ for low noise intensity.

**Fig 13:**
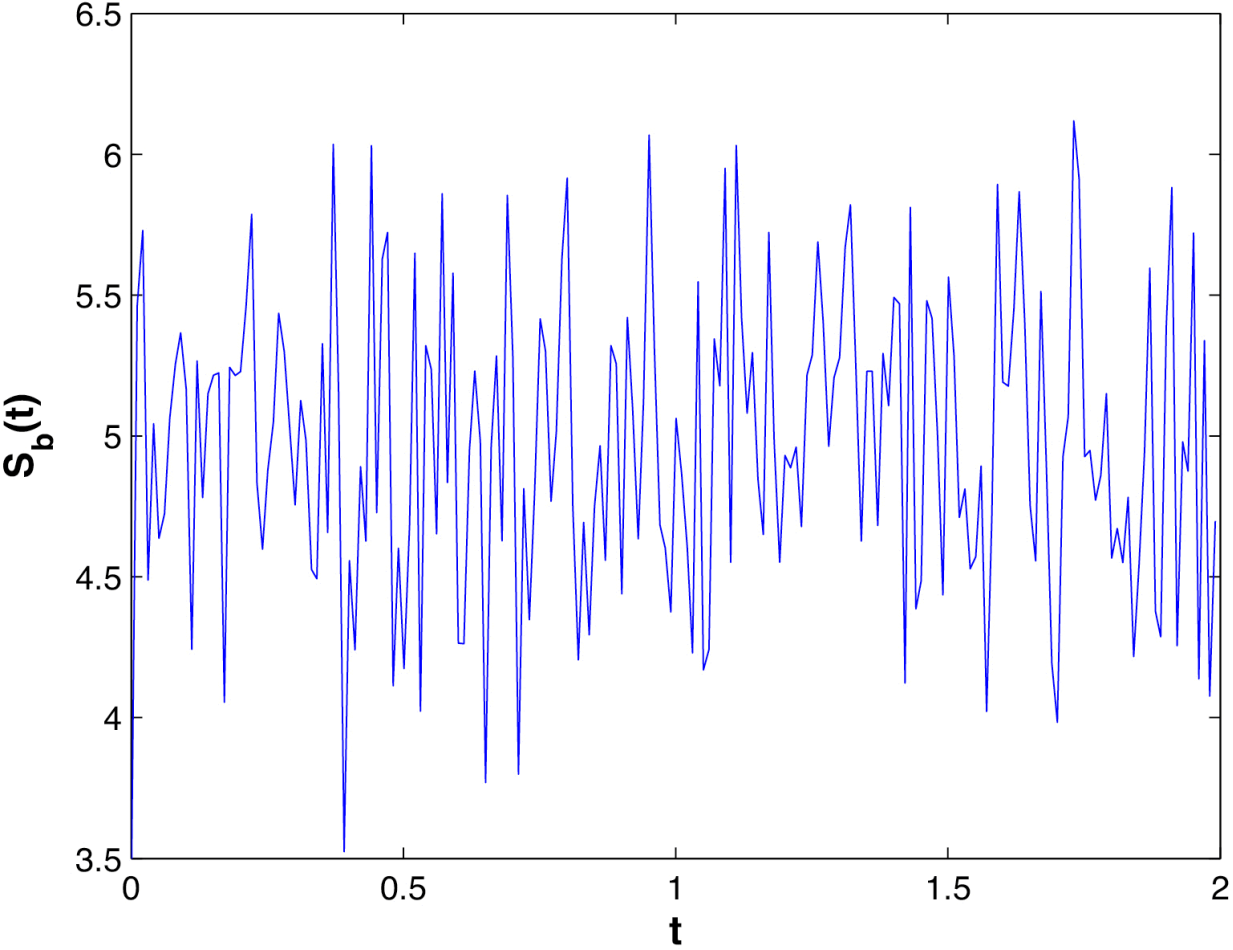
The evolution path of $S_{b}$ for low noise intensity.

**Fig 14:**
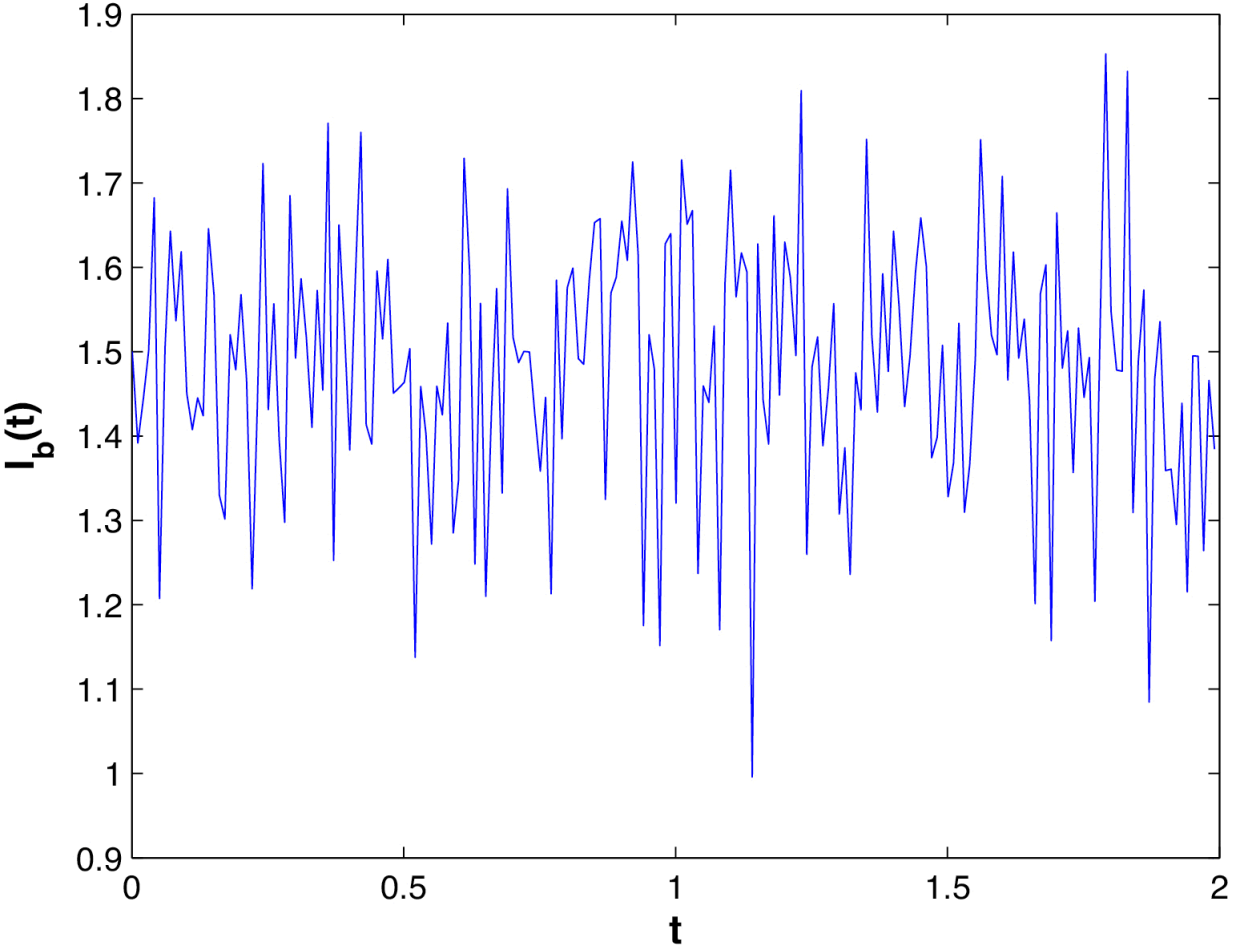
The evolution path of $I_{b}$ for low noise intensity.

**Fig 15:**
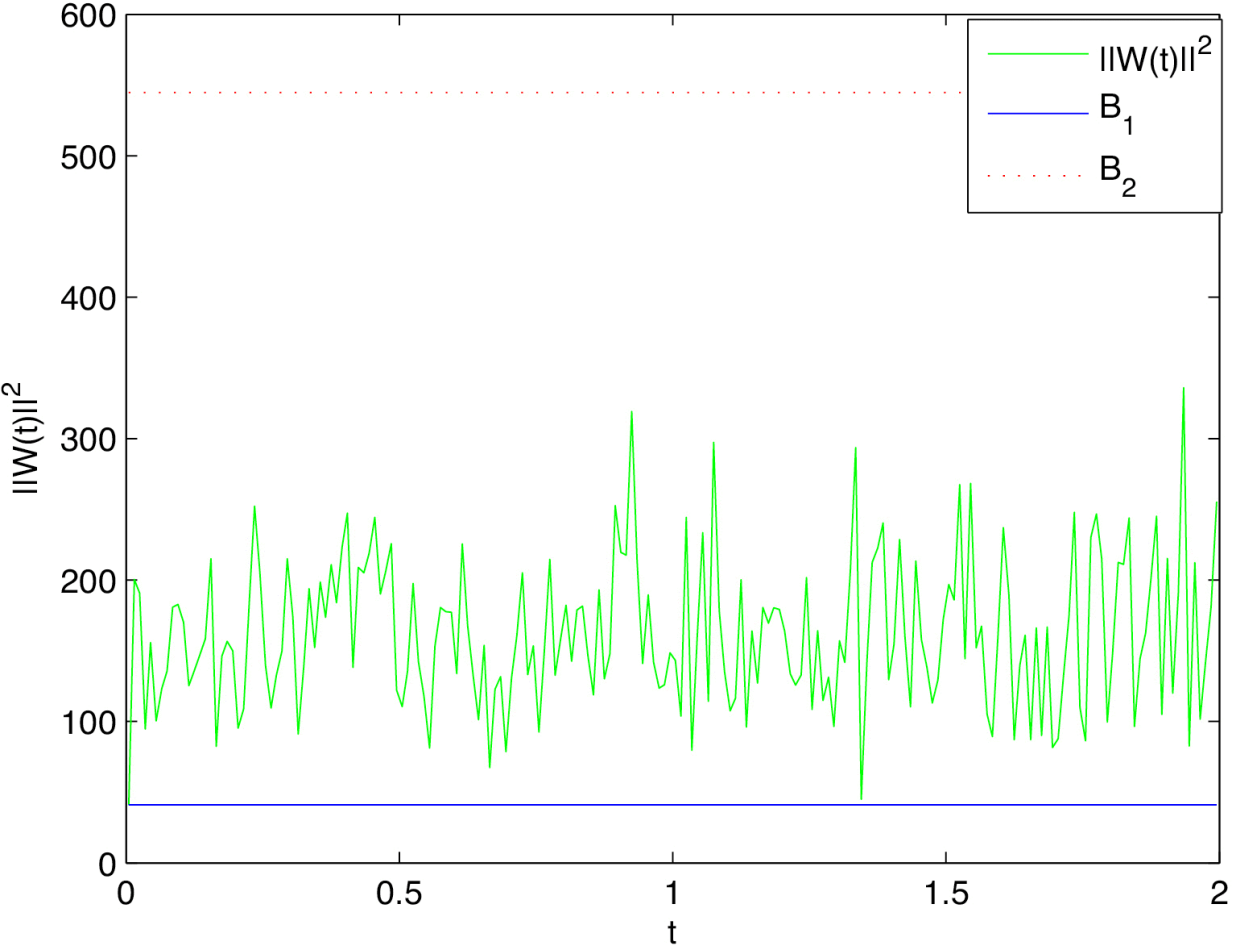
The state trajectories of system (1) for low noise intensity.

In Figs 11–15, let $\varepsilon_{1}=0.2$, $\varepsilon_{2}=0.1$, the evolution trajectories of $S_{m},I_{m},S_{b},I_{b}$ and the state trajectories of the norm *W* (*t*) are presented.

From Figs 1 – 4, 6 – 9 and 11 – 14, it can be observed that the system to attains a steady state more easily when the noise intensity is lower, whereas increased noise intensity makes stability more challenging to achieve. From Figs 5, 10 and 15, it can be observed that the system to attains a steady state more easily when the noise intensity is lower, whereas increased noise intensity makes stability more challenging to achieve. Through Figs 6 – 9, it can be seen that when the noise intensity increases, the solution will also increases, and at the same time, it can be observed from Fig 10 that the system does not satisfy finite-time stability either. Therefore, to effectively control the disease during an outbreak, external disturbances should be minimized as much as possible.

### The effect of mortality rates on finite-time stability

**Fig 16:**
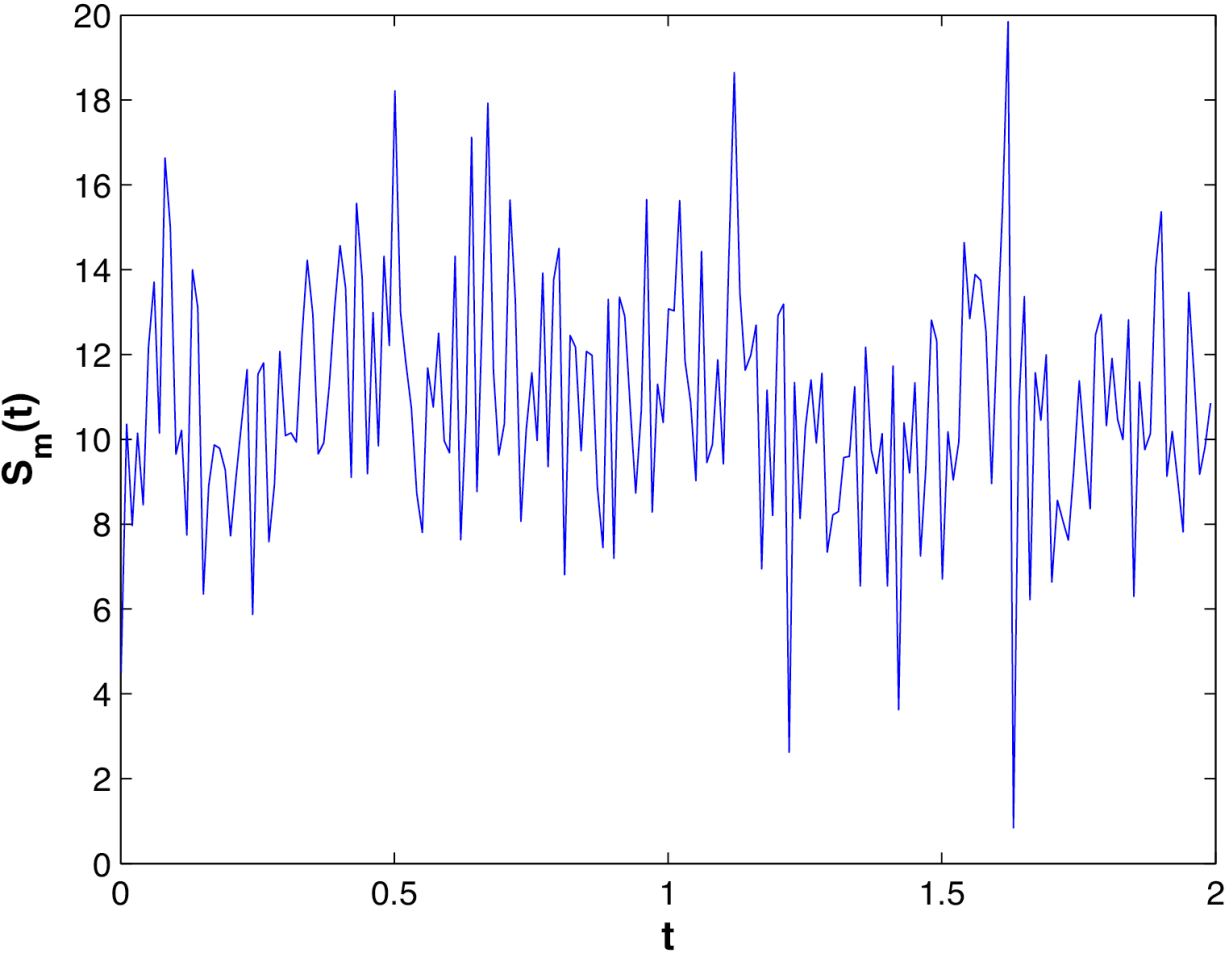
The evolution path of $S_{m}$ for low mortality rate.

**Fig 17:**
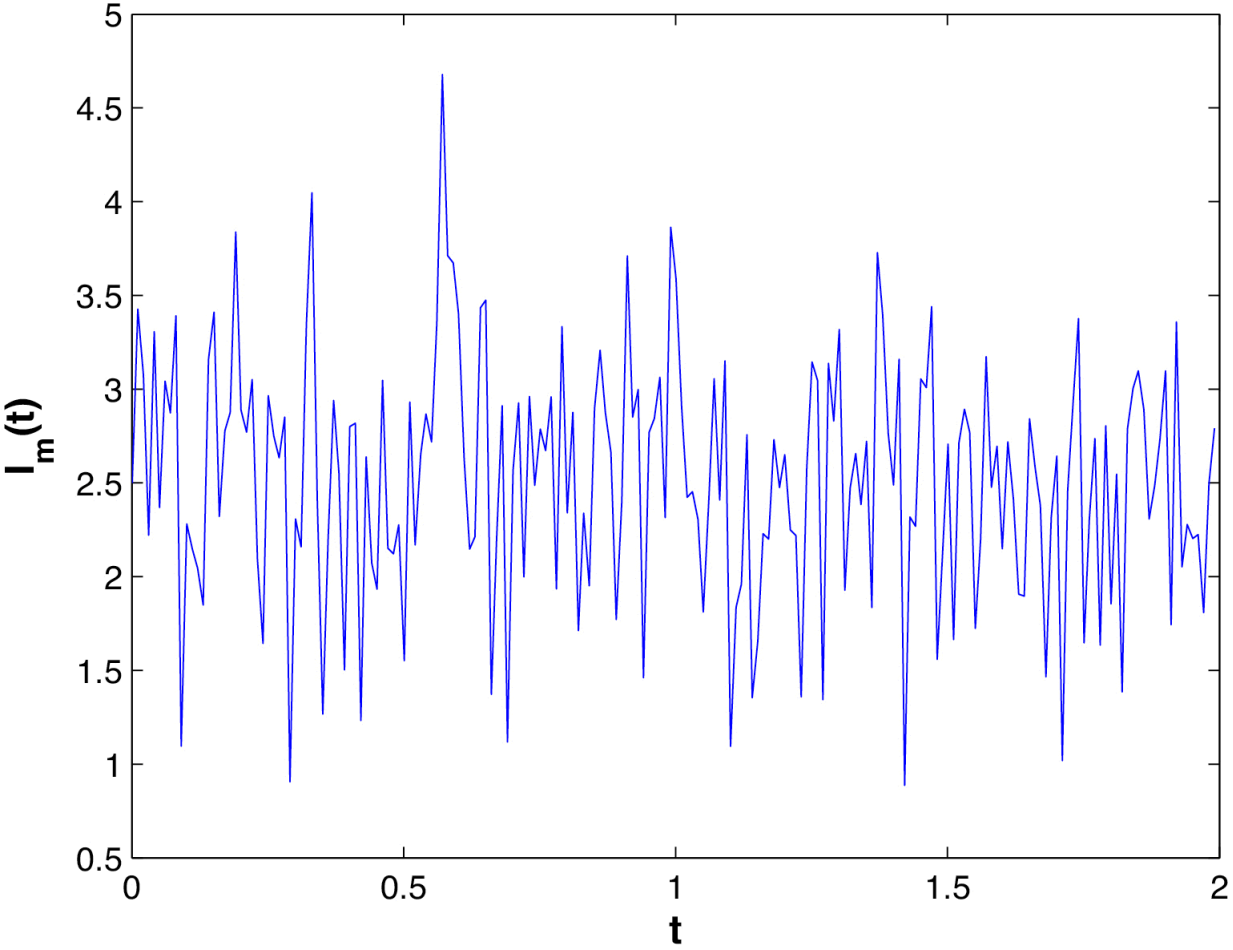
The evolution path of $I_{m}$ for low mortality rate.

**Fig 18:**
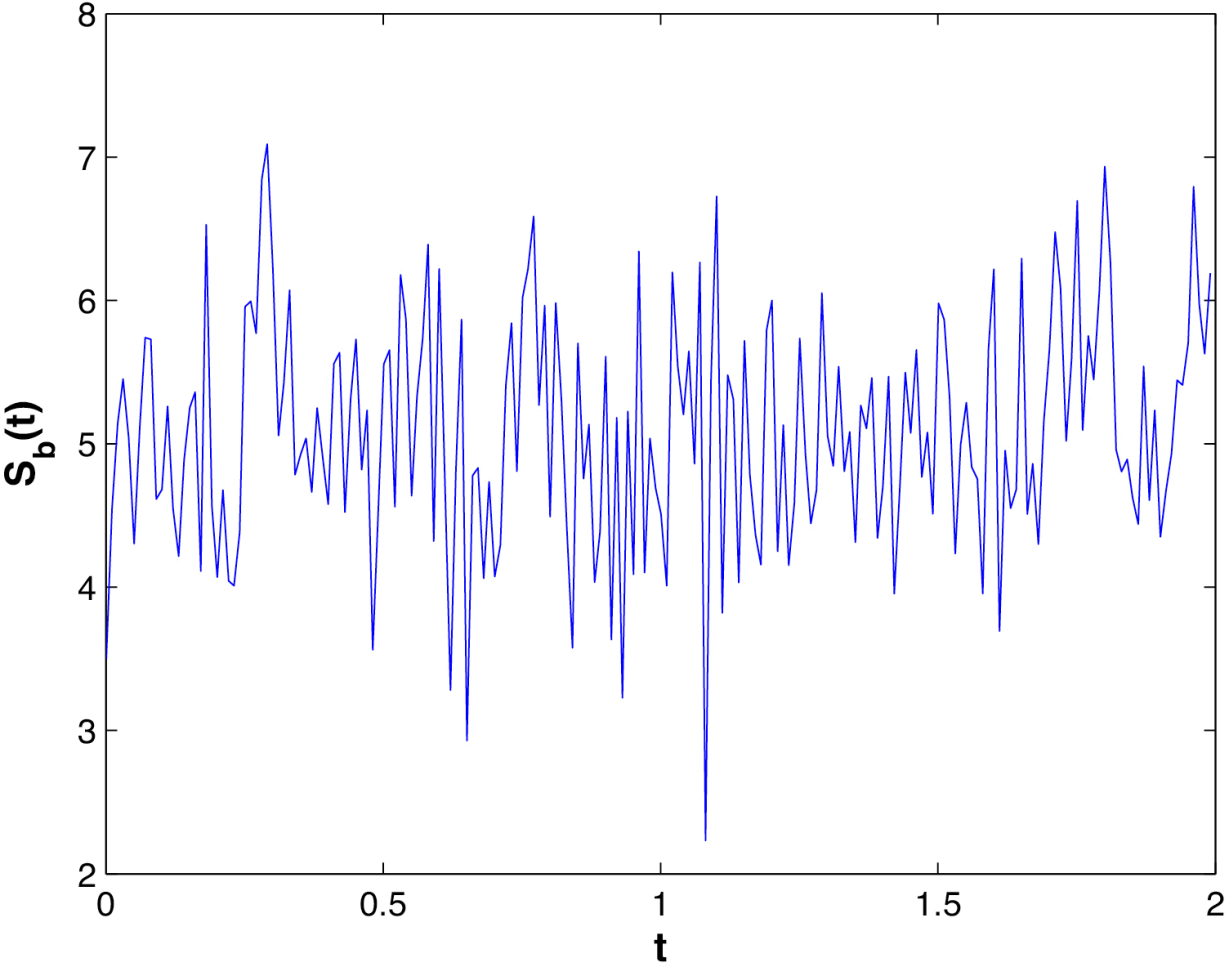
The evolution path of $S_{b}$ for low mortality rate.

**Fig 19:**
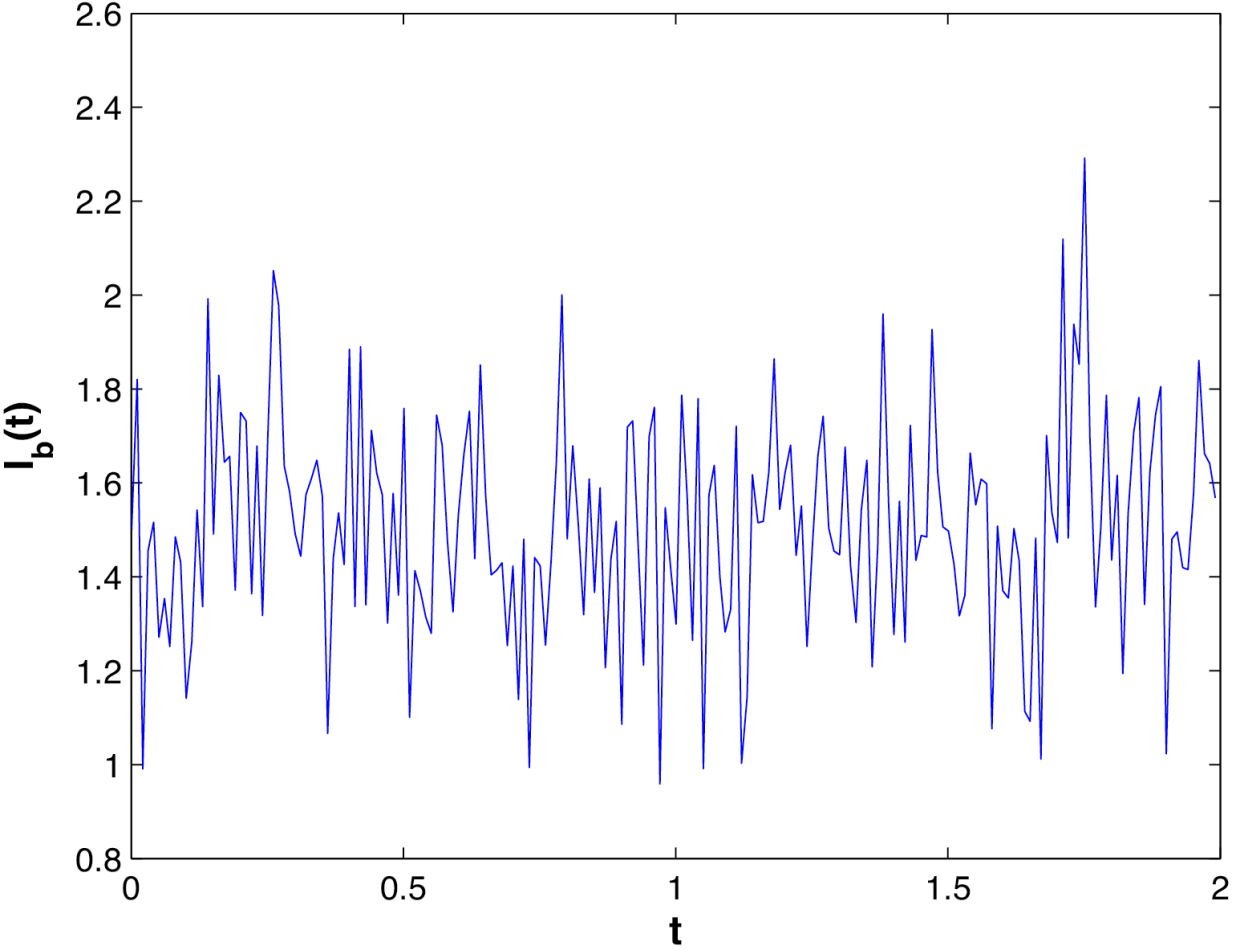
The evolution path of $I_{b}$ for low mortality rate.

**Fig 20:**
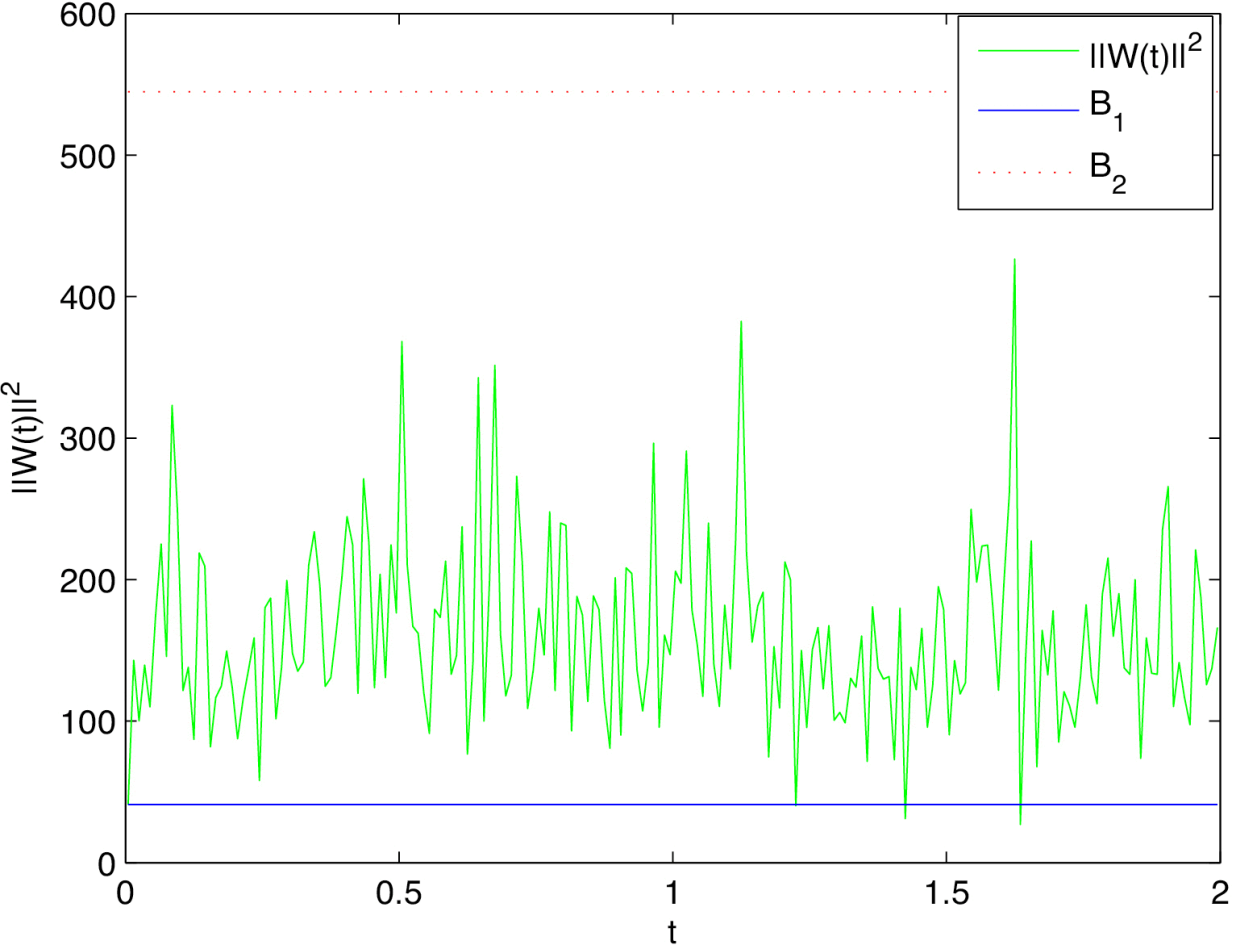
The state trajectories of system (1) for low mortality rate.

In Figs 16–19 and 20, Let $\mu_{m}=0.1$, $\mu_{b}=0.15$, $\varepsilon_{1}=0.25$, $\varepsilon_{2}=0.15$, and other parameters are as shown in Table 2. From Figs 1 – 4 and 16 – 19, it can be seen that both mosquito populations and bird individuals increase with the decrease of mortality rate. From Figs 5 and 20, reducing mortality rate leads to greater fluctuation intensity of the system’s state trajectory, and the trajectory will exceed the finite-time stability threshold at certain points.

### The effect of transmission rates on finite-time stability

**Fig 21:**
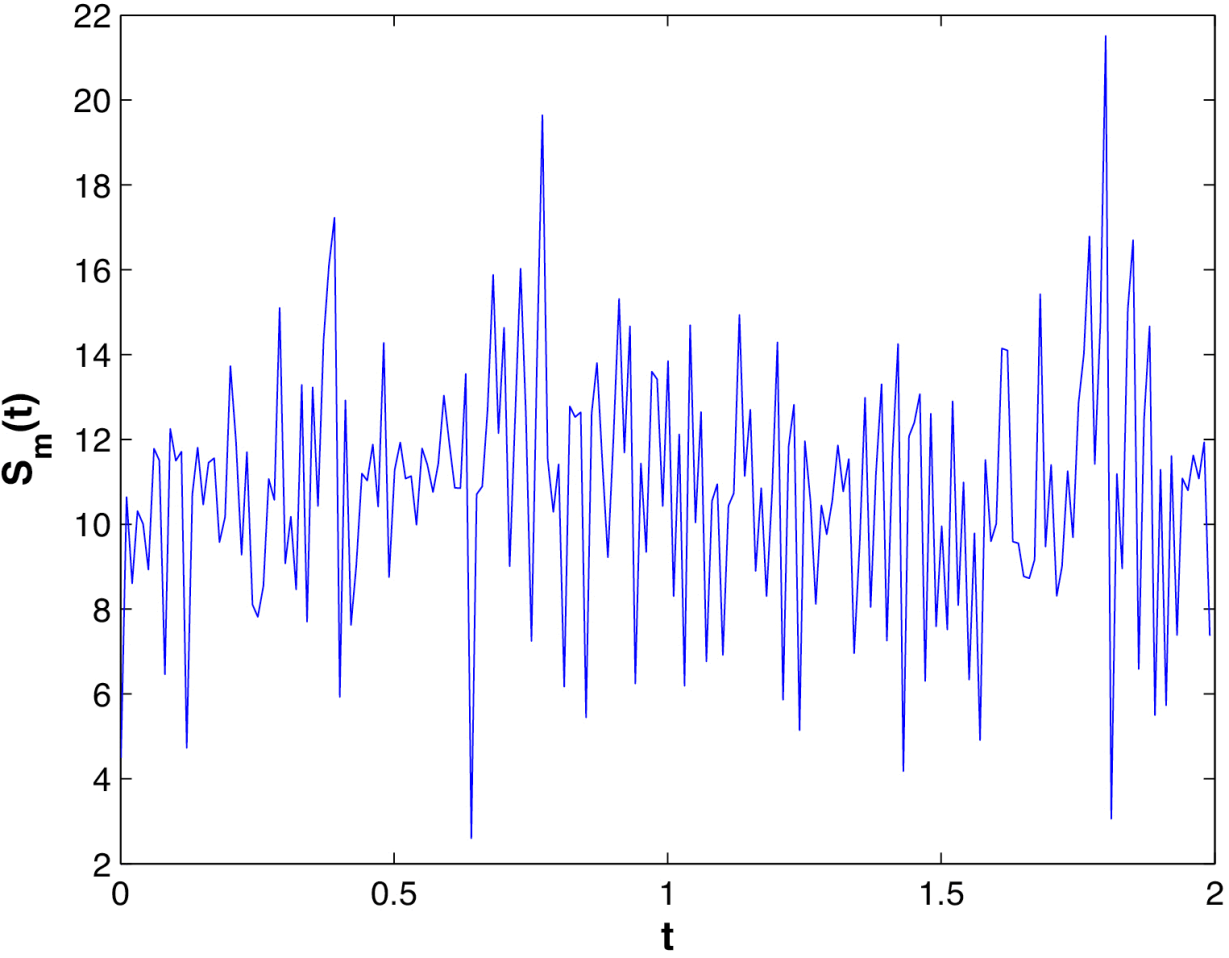
The evolution path of $S_{m}$ for high transmission rate.

**Fig 22:**
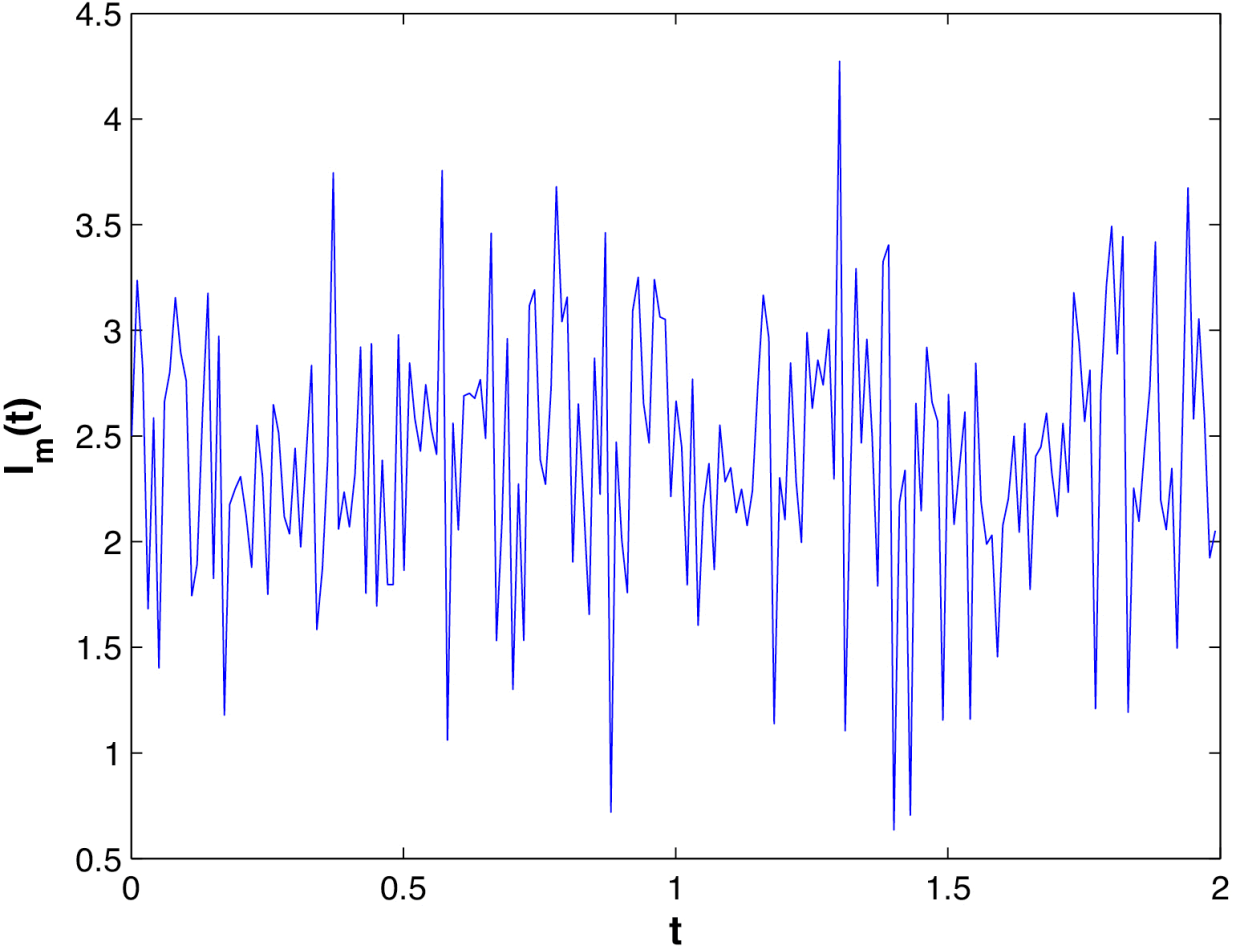
The evolution path of $I_{m}$ for high transmission rate.

**Fig 23:**
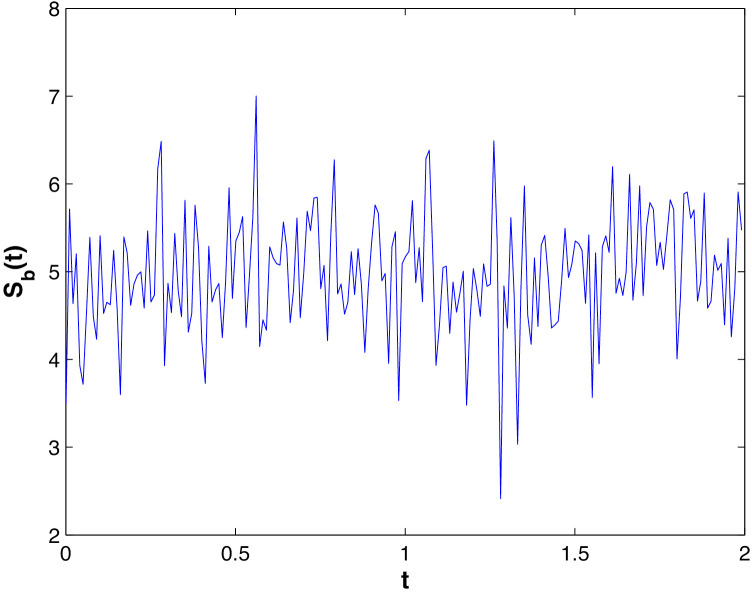
The evolution path of $S_{b}$ for high transmission rate.

**Fig 24:**
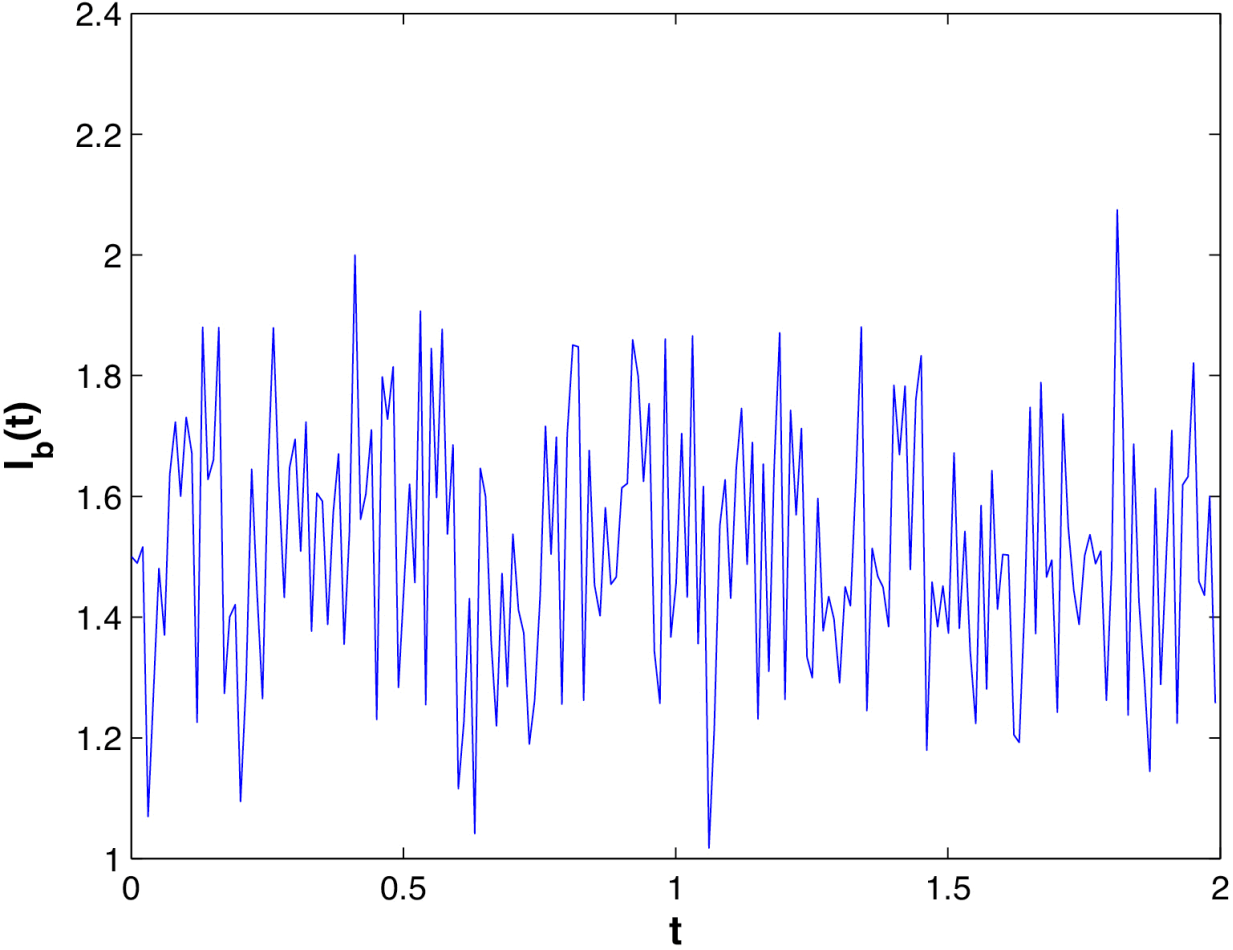
The evolution path of $I_{b}$ for high transmission rate.

**Fig 25:**
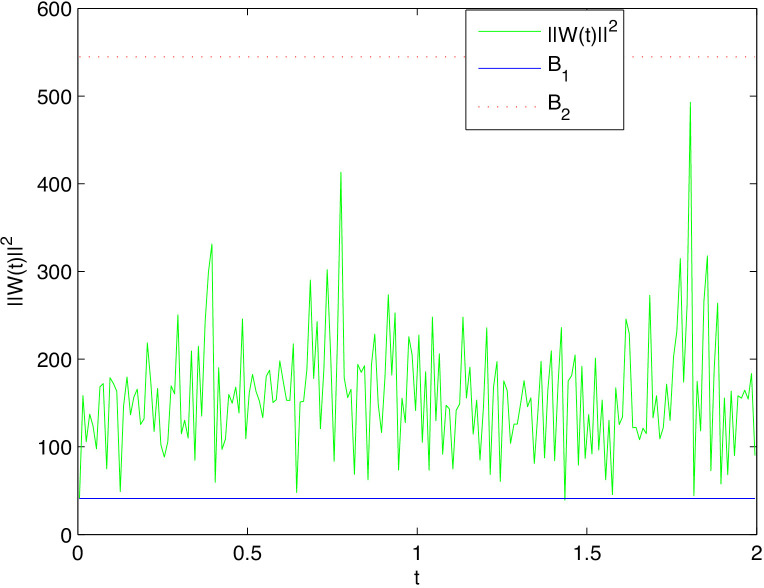
The state trajectories of system (1) for high transmission rate.

In Figs 21–24 and 25, let $\beta_{m}=0.8$, $\beta_{b}=1$, $\varepsilon_{1}=0.25$, $\varepsilon_{2}=0.15$, and other parameters are as shown in Table 2. As can be seen from Figs 5 and 25, with the increase of the transmission rate, the fluctuation amplitude of the state trajectories increases.

## 5. Conclusions

As the spread of the virus is highly influenced by external factors, particularly extreme weather conditions such as temperature, humidity, and rainfall, a stochastic West Nile virus model is established in this study. Initially, the boundedness, existence, and uniqueness of the solution are proven. Subsequently, by constructing a Lyapunov function, a sufficient condition or the finite-time stability of the West Nile virus model is derived. Finally, numerical simulations are conducted to validate the theoretical findings. Through these simulations, state trajectories under different noise intensities and transmission rates are analyzed, demonstrating that lower noise intensity facilitates the attainment of a steady state, whereas higher noise intensity makes stability more difficult to achieve, meanwhile, as the transmission rates increases, the fluctuation of the system’s state trajectory becomes greater. At the same time, reducing mortality rates leads to greater fluctuation intensity of the system’s state trajectory, and the trajectory will exceed the finite-time stability threshold at certain points. Therefore, based on the simulation results, to effectively control this disease, the first step is to reduce external interference, that is, to lower the noise intensity; Secondly, mosquito control and diseased poultry management should be implemented, and infected groups should be isolated or disinfected. Future research will focus on incorporating the effects of time delay, Lévy noise, and impulse perturbations on the finite-time stability of the system.
